# LC-MS/MS Characterization of Phenolic Metabolites and Their Antioxidant Activities from Australian Native Plants

**DOI:** 10.3390/metabo12111016

**Published:** 2022-10-24

**Authors:** Akhtar Ali, Jeremy J. Cottrell, Frank R. Dunshea

**Affiliations:** 1 School of Agriculture and Food, The University of Melbourne, Parkville, VIC 3010, Australia; 2 The Faculty of Biological Sciences, The University of Leeds, Leeds LS2 9JT, UK

**Keywords:** sandalwood nuts, wattle seeds, old man saltbush, lemongrass, medicinal plants, flavonoids

## Abstract

Polyphenols are considered vital bioactive compounds beneficial for human health. The Australian flora is enriched with polyphenols which are not fully characterized yet. Thus, the main objective of this study was to identify and characterize the Australian native sandalwood nuts, wattle seeds, lemongrass, and old man saltbush for phenolic compounds and their antioxidant activities. In this study, we tentatively identified a total of 155 phenolic compounds including 25 phenolic acids, 55 flavonoids, 22 isoflavonoids, 22 tannins, 22 lignans, 33 stilbenes, 33 coumarins and derivatives, 12 tyrosols and derivatives, and 6 phenolic terpenes. The highest total phenolic content (TPC) (15.09 ± 0.88 mg GAE/g) was quantified in lemongrass, while the lowest TPC (4.17 ± 0.33 mg GAE/g) was measured in wattle seeds. The highest total flavonoid content (TFC) and total condensed tannins (TCT) were measured in lemongrass and wattle seeds, respectively. A total of 18 phenolic metabolites were quantified/semi-quantified in this experiment. Lemongrass contains a vast number of phenolic metabolites.

## 1. Introduction

Australian native plants offer a substantial potential source of new antioxidant chemicals for use in medicines or functional products [1,2]. Because of their long history, the indigenous people in the area have contributed to ongoing improvements in our understanding of the characteristics and potency of numerous plants and food provenance [3]. The predominant plants in southeastern Australia are wattle trees (*Acacia* sp.), and the indigenous people use their seedlings as a staple diet. Wattle (*Acacia victoriae*) seeds are one of the commercially accessible local spices, and many people consider them to be a standard item in the culinary business. The toasted and pulverized seeds are used in baked products, mustards, flour mixes, sweet sauces, dressings, and drinks because of their ‘nutty’ taste [3]. They were suggested for incorporation in diabetic and other specialty diets because they are abundant in proteins and possess a lower glycemic index [4]. Triterpenes saponins extracted from *A. victoriae* seeds have been shown to reduce the growth of cancerous cells and prevent nuclear factor-kappa B (NFkB) activity [5]. The edible Acacia species’ seeds have proven to be both very nutrient-dense and safe to consume in both human clinical trials and laboratory investigations [4]. Wattle seeds are also identified as prickly wattle, gundabluie, elegant wattle, and bramble wattle. The flour of wattle seeds is pea-like flour and is also used in bread making. Due to its high protein profile, it has been consumed as a source of food in dairy products, seasonings, and bakery items for many years [4]. Multiple health advantages, including anti-cancer and anti-tumor properties, have been noted. There has not been much research into the content of phenolic and non-phenolic compounds in wattle seeds. Sandalwood (*Santalum spicatum*) nuts are precious gifts along with fragrant heartwood. These nuts are highly versatile with a unique flavor and amazing texture. Indigenous Australians used these nuts for the treatment of colds, stiffness, and body aches and to treat rheumatoid arthritis. Sandalwood nuts are also a rich source of protein (ca. 18%) and dietary fiber (ca. 17%), and contain more than 38% of omega-9 fatty acids. Old man saltbush (*Atriplex nummularia*) is an extremely versatile shrub widely used as a forage while the seeds are a traditional food source for many Aboriginal Australians. The earthy-flavored leaves of old man saltbush are edible and used for flavoring. Australian native lemongrass (*Cymbopogon ambiguous*) is an aromatic grass with a strong citrus flavor widely used as a medicinal herb to treat skin sores, chest infections, and flu symptoms by the Aboriginal people. Australians used to incorporate high amounts of these edible species into their daily diets. Many indigenous spices and herbs have gained recognition as beneficial components of Australian cuisine after entering into commercial production by bringing natural flavors and enhancing sensory properties [4]. 

Phytochemicals, including polyphenols, are the secondary plant metabolites abundantly found in fruits, vegetables, herbs, spices, and medicinal plants [6]. They show a positive impact on human and animal health when interacting with living tissues [7]. The compounds that possess an aromatic ring with at least one OH group are classified as phenolic compounds. Their structure can vary from simple phenols to complex polymers. More than 20,000 bioactive compounds have been identified in plants as phenolic compounds and among these more than 10,000 compounds have been identified as flavonoids [8]. Phenolic compounds are the diverse class of plant secondary metabolites including hydroxybenzoic acids and derivatives, cinnamic acids and derivatives, flavonoids, isoflavonoids, neoflavonoids, tannins, lignans, stilbenes, coumarins, curcuminoids, phenolic terpenes, tyrosols, xanthones, simple phenols, and other polyphenols [9]. In polyphenols, flavonoids (anthocyanins, flavanols, flavonols, flavanones, flavones, chalcones, and dihydroxy chalcones) are the most abundant phenolic compounds in nature [10]. There has not been much research into the content of phenolic and non-phenolic compounds in these selected Australian native plants. Australian native plants are a promising source of antioxidants, nutraceuticals, and functional foods. An excessive production of free radicals in the body can trigger different pathological conditions. Therefore, the inclusion of antioxidants in the daily diet is important to deactivating the free radicals in the body [11]. 

The emerging interest in the food supply required detailed analytical characterization and quantification of antioxidants to collectively understand their role in food and human health [11]. Previously, limited studies were conducted to explore the phenolic metabolites in selected Australian native plants. The identification and characterization of phenolic compounds in these plants could further explore the use of these plants in the food, feed, and pharmaceutical industries. Therefore, the main aim of this study was the identification, characterization, and quantification of phenolic metabolites in Australian native lemongrass, wattle seeds, old man saltbush, and sandalwood nuts for phenolic compounds and their antioxidant potential. In this context, we measured total phenolic content (TPC), total flavonoid content (TFC), total condensed tannins (TCT), and their antioxidant activities including 2,2′-azinobis-(3-ethylbenzothiazoline-6-sulfonic acid (ABTS), ferric reducing antioxidant power (FRAP), hydroxyl-radical scavenging activity (^•^OH-RSA), ferrous ion chelating assay (FICA), 2,2′-diphenyl-1-picrylhydrazyl (DPPH) and phosphomolybdate assay (PMA) were quantified in selected Australian native plants. Moreover, LC-ESI-qTOF-MS/MS was used for the screening and characterization of phenolic compounds from these Australian plants. This study will explore the use of native Australian plants at the industrial level.

## 2. Materials and Methods

### 2.1. Chemicals and Reagents

Sigma-Aldrich Aldrich (Castle Hill, NSW, Australia) provided most of the chemicals utilized for extraction and characterization. Gallic acid, Folin–Ciocalteu’s phenol reagent, L-ascorbic acid, sodium phosphate, vanillin, aluminum chloride hexahydrate, iron(III) chloride hexahydrate (FeCl_3_·6H_2_O), sodium phosphate monobasic monohydrate, sodium phosphate dibasic heptahydrate, trichloroacetic acid, hydrochloric acid, ethylenediaminetetraacetic acid (EDTA), ferrozine, ammonium molybdate, 3-hydroxybenzoic acid, catechin, iron (II) sulfate heptahydrate, potassium ferrocyanide(III), DPPH, 2,4,6 tripyridyl-s-triazine (TPTZ), and ABTS were purchased from Sigma Aldrich (Castle Hill, NSW, Australia). From Chem-Supply Pty Ltd. (Adelaide, SA, Australia), we purchased sodium carbonate anhydrous and hydrogen peroxide (30%), and we also purchased 98% H_2_SO_4_ from RCI Labscan (Rongmuang, Thailand). Thermo Fisher Scientific Inc. supplied methanol, ethanol, acetonitrile, formic acid, glacial acetic acid, iron (III) chloride anhydrous, and HPLC and LC-MS-grade chemicals (Scoresby, VIC, Australia). Thermo Fisher Scientific provided 96-well plates for various in vitro bioactivities and antioxidant tests (Scoresby, VIC, Australia). HPLC vials (1 mL) were also purchased from Agilent equipment (Melbourne, VIC, Australia).

### 2.2. Extraction and Preparation of Samples

Australian native wattle seeds were purchased from Natif (www.natif.coma.au, accessed on 21 September 2021), native lemongrass from Tucker Bush (www.tuckerbush.com.au, accessed on 21 September 2021), while old man saltbush and sandalwood nuts were purchased from Australian super Foods (www.australiansuperfoods.com.au, accessed on 21 September 2021). Wattle seeds and sandalwood nuts were crushed and dried at 50 °C in the oven for 4 days and again ground and defatted with *n*-hexane before the phenolic extraction. Phenolic compounds were extracted by following the method of Ali et al. [11] in triplicate. 

### 2.3. Measurement of TPC, TFC, and TCT

The TPC, TFC, and TCT of Australian native plants were measured by following the methods of Ali et al. [11], Zahid et al. [12] and Ali et al. [13] while all experiments were conducted in triplicate. 

### 2.4. Measurement of Antioxidant Activities 

The DPPH of selected plant extracts was measured by following the method of Zahid et al. [14] while the ABTS value of selected plants was quantified using the method of Bashmil et al. [15] in triplicate. The FRAP was measured by following the method of Ali et al. [13]. The ^•^OH-RSA of plant extracts was measured by following the method of Chou et al. [16] with modifications. To do this, 50 μL of plant extract, 50 μL 6 mM aqueous solution of FeSO_4_. H_2_O, and 50 μL 6 mM H_2_O_2_ solution in water were mixed and incubated at 25 °C for 20 min. After that, 50 μL of 3-hydroxybenzoic acid 6 mM solution in water was added and again incubated for 20 min before the absorbance reading at 510 nm. Ascorbic acid (0–300 μg/mL) in Milli-Q water was used as a reference standard to generate the equation. The PMA of plant extracts was measured by following the method of [13] with minor modifications. Briefly, 40 μL of plant extracts was mixed in 260 μL phosphomolybdate dye (0.6 M H_2_SO_4_ in H_2_O, 28 mM trisodium phosphate solution in H_2_O and 4 mM ammonium molybdate solution in water were mixed in a ratio of 1:1:1 (*v*/*v*) to make phosphomolybdate dye) and incubated at 90 °C for 90 min in a water bath after properly wrapping the 96-well plates in aluminum foil. Then, the plates were cooled, and absorbance was recorded at 695 nm, while ascorbic acid (0–200 μg/mL) was used to generate a standard curve. The FICA of plant extracts was measured by following the method of [11]. 

### 2.5. LC-ESI-QTOF-MS/MS Characterization and Semi-Quantification of Phenolic Metabolites 

The methods of Ali et al. [11], Suleria et al. [17], and Ali et al. [8] were used to identify and characterize phenolic metabolites from Australian native lemongrass, wattle seeds, old man saltbush, and sandalwood nuts. A Synergi 4 μm Hydro-reversed phase (RP 80 Å) LC column (250 × 4.6 mm) connected with C18 ODS (4.0 × 2.0 mm) guard column was used in this experiment. Agilent 6520 Accurate Mass QTOF LC-MS/MS equipped with an Agilent 1200 HPLC (Agilent Technologies, Santa Clara, CA, USA) was used for the identification of unknown phenolic metabolites from Australian native plants. Briefly, an aliquot of 10 μL from each plant extract was injected with a flow rate of 0.6 mL/min with a following gradient: 10 min (10–20 % B), 10–20 min (20–25% B), 20–30 min, (25–30% B), 30–40 min (30–45% B), 40–50 min (45–60% B), 50–65 min (60–90% B), 65–67 min (90–100% B), 67–68 min (100–10% B), and 68–70 min (10% B) of mobile phase A (0.1% LC-MS grade formic acid in Milli-Q water) and mobile phase B (0.1% LC-MS grade formic acid in acetonitrile). Furthermore, all other settings were used as described by Ali et al. [11]. Agilent MassHunter Workstation Software Quality Analysis (version B.06.00) was used for the identification and characterization of phenolic metabolites with the help of the Personal Compounds Database and Library (PCDL) for metabolites, PubChem (https://pubchem.ncbi.nlm.nih.gov, accessed on 21 September 2021), Human Metabolome Database (https://hmdb.ca, accessed on 21 September 2021), and FooDB (https://foodb.ca), accessed on 10 May 2022 while offline versions of GNPS, NIST, and MassBank libraries and databases were also used in this experiment to match the MS/MS spectra of phenolic metabolites. All the samples were repeated in duplicate and a total of 18 phenolic compounds were semi-quantified in this experiment. MS/MS spectra of 40 commercial standards were also acquired in this experiment. A mixture of 24 commercial standards was used to generate equations through LC-MS/MS in duplicate.

### 2.6. Statistical Analysis

XLSTST-2019.1.3 was used for biplot analysis while the Minitab Program for Windows version was used for a one-way analysis of variance (ANOVA) followed by Tukey’s honestly significant test.

## 3. Results and Discussion

### 3.1. Measurement of Total Polyphenols (TPC, TFC, and TCT)

Phenolic metabolites are vital for human health and are widely present in fruits and medicinal plants [9]. The screening and characterization of polyphenols has attracted much attention due to their wide use in the food, feed, pharmacological, and medicinal industries. The results for the measurement of total phenolic content (TPC), total flavonoid content, and total condensed tannins (TCT) are given in Table 1.

The TPC represents phenolic acids, flavonoids, isoflavonoids, lignans, stilbenes, and other polyphenols. In this experiment, the highest TPC (15.09 ± 0.88 mg GAE/g) was measured in native lemongrass while the lowest TPC (4.17 ± 0.33 mg GAE/g) was measured in wattle seeds. The TPC of lemongrass was comparable to basil, thyme, bay, and nutmeg while the TPC of wattle seeds, sandalwood nuts, and old man saltbush was comparable to black cumin, black cardamom, cumin, fennel, black pepper, dill, parsley, and fenugreek [9,13]. Previously, Ee et al. [18] studied roasted and raw wattle seeds, and total polyphenols were measured from 3.53 ± 0.05 to 12.19 ± 0.37 mg GAE/g. Meanwhile, Hannachi et al. [19] reported an average of 6.32 mg GAE/g in wattle seeds from Tunisia. Furthermore, Konczak et al. [4] measured 0.8 ± 0.12 mg GAE/g total phenolics in Australian native wattle seeds, while Sommano et al. [20] reported 2.65 mg GAE/g total phenolics in Australian native wattle sees. Previously, Irfan et al. [21] measured total phenolic contents in lemongrass from Pakistan in the range of 32.9 to 61.2 mg GAE/g in acetone and ethanol extracts, while Godwin et al. [22] measured total phenolic content in lemongrass in the range of 1.3 to 7.3 mg GAE/g in cold and hot water. Moreover, Juntachote et al. [23] measured the total phenolic content in lemongrass in the range of 0.53 to 1.0 mg GAE/g in ethanolic fractions. The TPC of old man saltbush and wattle seeds is comparable to mountain pepper (5.91 ± 0.32 mg GAE/g) and tamarind (3.72 ± 0.12 mg GAE/g), respectively, as reported by Cáceres-Vélez et al. [24]. The variation in total phenolics reflects the diversity of phenolic compounds and their ability to reduce the F-C reagent. Moreover, the variations in total phenolics can be attributed to different extraction conditions in the current or later studies, type of solvent, solvent concentrations, solvent-to-sample ratio, time and temperature combinations, and geographical locations where these Australian native plants were grown [9,13]. 

Flavonoids are the vital and the most abundant plant secondary metabolites found in fruits, herbs, and medicinal plants. More than 10,000 flavonoids have been discovered in nature [8]. The highest TFC (3.07 ± 0.08 mg QE/g) was quantified in lemongrass while the lowest TFC (0.67 ± 0.05 mg QE/g) was in wattle seeds. The TFC of sandalwood nuts and old man saltbush was measured at 2.81 ± 0.21 mg QE/g and 2.32 ± 0.12 mg QE/g, respectively. Interestingly, the highest TCT (2.88 ± 0.10 mg CE/g) was measured in wattle seeds while the lowest TCT (1.12 ± 0.06 mg CE/g) was measured in sandalwood nuts. Previously, Ee et al., [18] measured the total flavonoid content of wattle seeds in the range of 0.23 to 1.93 mg CE/g under different roasting conditions. The flavonoids in wattle seeds are comparable to lentils, soy, common beans, and kidney beans which contained total flavonoids in the range of 0.85 to 1.14 mg QE/g [18,25]. The TFC of lemongrass was found comparable to flavonoids in cumin, basil, and dill while the value of sandalwood nuts and old man saltbush was found comparable to flavonoids in bay leaf, fenugreek, and black cumin [9,13]. Previously, a limited number of studies have been conducted to measure the total flavonoid content in these selected plants. The variations in total flavonoid content in the current and later studies may be attributed to the different factors mentioned above. Moreover, proper screening, identification, and characterization with LC-MS/MS can provide more reliable information regarding the presence of individual phenolic metabolites in these selected Australian native plants. 

### 3.2. Antioxidant Potential of Australian Native Plants

**Figure 1:**
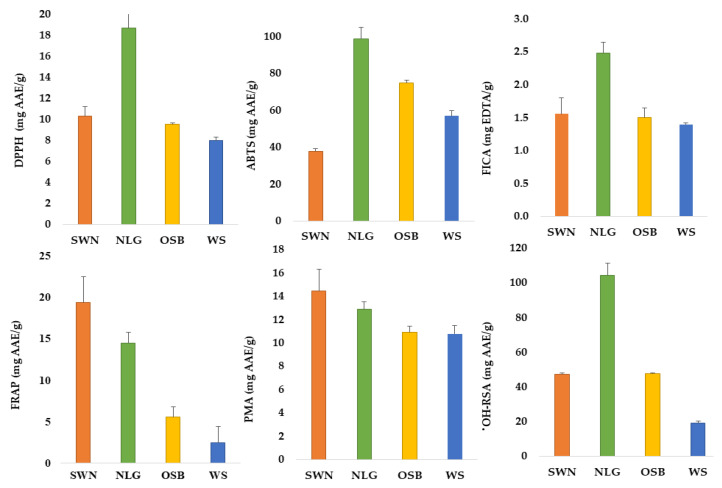
Antioxidant activities of Australian native lemongrass (LG), sandalwood nuts (SWN), wattle seeds (WS), and old man saltbush (OSB).

Antioxidants are the chemical constituents in the human diet which are capable of deactivating the free radicals in the human body and these antioxidants are obtained from herbs, spices, fruits, and vegetables. In this study, the antioxidant potential of Australian native plants was measured through DPPH, ABTS, FICA, FRAP, PMA, and ^•^OH-RSA. The results of quantified antioxidant activities are given in Figure 1 and Table S1.

DPPH is a low-cost assay and is used to estimate the ability of samples to scavenge the free radicals in biological systems as it works based on the ability to donate electrons or hydrogen ions. DPPH is a free radical which contains stable nitrogen in its center and reduces its bluish-purple color when mixed with the extract of selected plants. These are known as radical scavengers as any substance that causes this reaction can be categorized as an antioxidant. Table S1 indicates that the DPPH for lemongrass (18.73 ± 2.8 mg AAE/g) and sandalwood nuts (10.30 ± 0.9 mg AAE/g) were higher (*p* < 0.05) than other selected plants. As the flavonoid content of lemongrass was higher, this could be the reason for its higher DPPH. Many studies have reported that the radical scavenging activity of lemongrass is higher than that of many other medicinal plants. The ABTS is also a widely used assay for estimating the free radical scavenging capacity of plant extracts, including hydrophilic and lipophilic constituents, based on the polyphenols’ hydrogen ion donating ability. ABTS^+^ radical cation inhibition is based on the characteristic wavelength which is 734 nm [26]. The ABTS values of lemongrass (98.81 ± 6.19 mg AAE/g) and old man saltbush (74.76 ± 1.61 mg AAE/g) were estimated to be higher than those of other selected plants, while the lowest ABTS value was found in sandalwood nuts (37.72 ± 1.40 mg AAE/g). 

The functional group of iron used in the biological system is responsible for the ability of iron chelation in Australian native plants. The highest value of FICA was found in lemongrass (2.48 ± 0.24 mg EDTA/g) compared to other selected plants on the list. Lipid peroxidation is responsible for catalyzing and FICA reduces the concentration of transition metals, which makes FICA a vital component. Chelating agents stabilize the metal ions’ oxidized form by forming s-bonds with metal, and in this way, the redox potential is reduced. Ferrous ions can increase lipid peroxidation by Fenton’s reaction and this process is carried out by dismantling the lipid peroxides and hydrogen to free radicals. When ferrous ion decomposes lipid hydroperoxides into alkoxyl and peroxyl radicals, lipid peroxidation is increased. In this reaction, a complex bond is formed between ferrozine and ferrous ion, and the herbal extracts resist this complex formation. In this way, the herbal extracts minimize ferrous ions and protect against oxidative damage. 

The Fe^+3^–TPTZ complex reducing the ability of antioxidant compounds to Fe^+2^–TPTZ complex in the biological system was evaluated through the FRAP assay [9,13]. The results showed that sandalwood nuts and lemongrass have significantly higher FRAP than the other selected Australian native plants (*p* < 0.05). The highest FRAP was found in sandalwood nuts (19.48 ± 3.04 mg AAE/g) and lemongrass (14.55 ± 1.32 mg AAE/g) while wattle seeds were found with the lowest FRAP (2.52 ± 1.97 mg AAE/g). Previously, A positive correlation of flavonoids with antioxidant activities indicated that flavonoids are the main antioxidant constituents [26]. Australian native plants can contain different reducing agents which can bind with free radicals to terminate or stabilize the chain reactions in the biological systems [27]. Thus, the higher reduction power of selected Australian plant extracts indicates their higher antioxidant capacity. The reduction capacity of molybdenum (VI) to molybdenum (V) is measured by using the Phosphomolybdenum antioxidative power assay (PMA assay). This process is performed with an antioxidant phenolic compound followed by the formation of a green molybdenum (V)/phosphate complex. It is indicated from the results that sandalwood nuts have higher PMA (14.43 ± 1.86 mg AAE/g) than other selected Australian native plants.

The anti-radical capacity of selected Australian native plants was also measured by using ^•^OH-RSA. The highest value of ^•^OH-RSA was found in lemongrass (104.34 ± 6.92 mg AAE/g) while the minimum value was found in wattle seeds (19.25 ± 0.92 mg AAE/g). Hydroxyl radicals (^•^OH) are one of the most reactive species that are involved in DNA damage, lipid peroxidation, and biological damage by attacking each molecule found in the biological system. Protection from biological damage against free radicals could be prevented by the scavenging of ^•^OH radicals. 

It is reported that antioxidant activities vary in selected Australian native plants due to their complex mixture of bioactive compounds, and mainly depend on the method used for extraction. To determine the antioxidant potential of plants, there is a list of methods with their benefits and limitations [15,28]. Due to the complex nature of phenolic compounds and multiple mechanisms of reactions in the biological system, no defined method truly reflects the same antioxidant potential of these bioactive compounds [29]. Various studies have been conducted to estimate the antioxidant activities of different plants from different geographical locations [30,31,32,33,34,35] but studies on Australian native plants are limited. Total polyphenols in Australian native plants and their antioxidant capacities demonstrate that further research is needed to identify and verify the actual contribution of polyphenols towards antioxidant potential while eliminating or minimizing the contribution of non-phenolic metabolites.

### 3.3. Pearson Correlation and Biplot Analysi of Phenolic Contents and Antioxidant Activities

A Pearson correlation analysis was conducted between phenolic contents and antioxidant activities of Australian native plants given in Table 2. 

**Figure 2:**
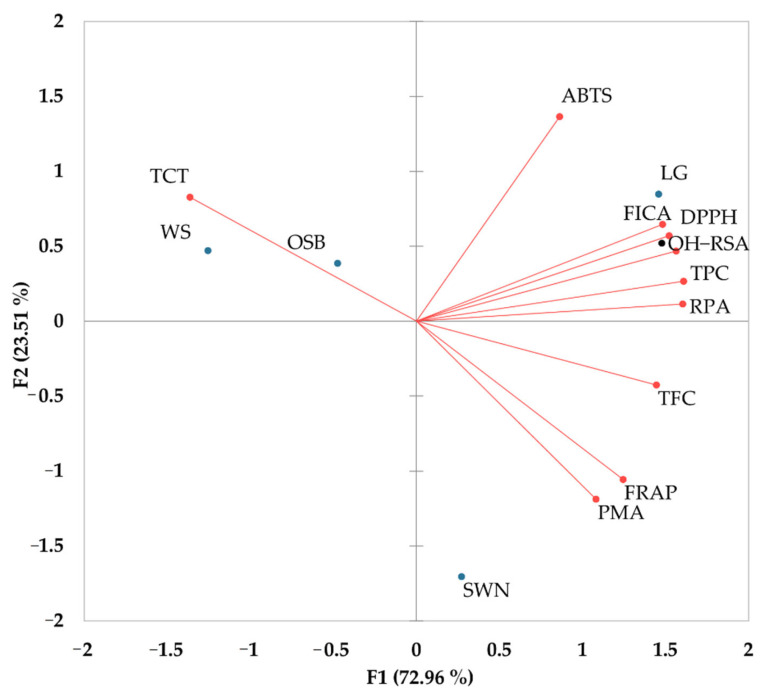
Biplot analysis of phenolic contents and their antioxidant activities in Australian native plants.

It indicates that a highly significant correlation of TPC was observed with DPPH (*r* = 0.98), RPA (*r* = 0.99), FICA (*r* = 0.97), and ^•^OH-RSA (*r* = 0.98) while TCT negatively correlated with other antioxidant activities (Table 2). These results indicate that mainly total phenolic content and total flavonoid content in selected Australian native plants are responsible for these antioxidant activities. The variation in antioxidant activities indicates the diversity of phenolic and non-phenolic compounds in these selected Australian native plants. It has been established that the antioxidant potential of flavonoids depends on the availability of an OH-group on the ring B and whether it can donate electrons or hydrogen atoms to a free radical in a biological system [11]. Furthermore, the mechanism of antioxidant reactions, experimental conditions, and the synergistic/antagonistic reactions of different compounds in the extract can affect the antioxidant activity and relationship with total phenolic and flavonoid contents [8,9]. In addition, a biplot analysis further elaborates the correlation between selected Australian native plants, phenolic contents, and their antioxidant activities (Figure 2). It depicts that F1 has a higher contribution (72.96%) than F2, which has a lower contribution (23.51%) to the antioxidant activities of Australian native plants. Both components (F1 and F2) explained the total variability (96.47%) in these selected antioxidant activities of selected plants. Additionally, it indicates that a higher amount of total condensed tannins in wattle seeds and old man saltbush negatively correlated with the PMA, FRAP, and TFC. Overall, the TCT value did not observe any correlation with the other antioxidant activities. Moreover, the higher concentration of total phenolic content in lemongrass is observed a strong positive correlation with the RPA, ^•^OH-RSA, DPPH, FICA, and ABTS activities while the higher concentrations of flavonoid contents in sandalwood nuts are positively correlated with the PMA, FRAP and RPA activities. The structure of flavonoids significantly affects antioxidant reactions. The presence of more OH-groups in flavonoids is favorable for antioxidant reactions, while antioxidant activity will also increase if the C3–C4 position in the ring B is replaced with OH-groups [11]. 

### 3.4. LC-MS/MS Identification of Bioactive Phenolic Metabolites from Australian Native Plants

The untargeted screening and characterization of phenolic metabolites in Sandalwood nuts, native lemongrass, old man saltbush, and wattle seeds were identified and characterized by using LC-ESI-QTOF-MS/MS, and MS/MS spectra were compared with libraries and published literature to confirm the phenolic metabolites (Figures S1 and S2). A total of 155 phenolic metabolites were tentatively identified in these selected Australian native plants (Table 3). 

#### 3.4.1. Phenolic Acids

Phenolic acids (hydroxybenzoic acids and hydroxycinnamic acids) are widely present in plants [36]. Their main applications are in cosmetics, medicinal industries, health, and pharmacology due to their antioxidant, anti-aging, and anti-microbial properties [37]. These are aromatic secondary metabolites that have health benefits. In this study, a total of 33 phenolic acids (7 hydroxybenzoic acids, 24 hydroxycinnamic acids, and 2 hydroxyphenyl acetic acids) were characterized by MS/MS which was used for the confirmation of their fragmentation patterns (Table 3). The fragmentation pattern of phenolic acids generally is shown by the removal of carbon dioxide and hexosyl moiety from their parent ions.

##### Benzoic Acids and Their Derivatives

Benzoic acids and derivatives are also called benzenoids and are widely present in plants. A total of seven hydroxybenzoic acids were tentatively identified in these Australian native plants. Compounds 1 (protocatechuic acid), 2 (gallic acid), and 4 (*p*-hydroxybenzoic acid) produced fragment ions at *m*/*z* 109, *m*/*z* 125, and *m*/*z* 93 after the loss of CO_2_ (44 Da) from the precursor ions, respectively [9,13]. The compounds 3 (protocatechuic acid 4-*O*-glucoside) and 7 (punicalin) were only identified in lemongrass and old man saltbush, researchers. Protocatechuic acid 4-*O*-glucoside (compound 3-C_13_H_16_O_9_) was putatively characterized in lemongrass at ESI^−^ *m*/*z* 315.0735, which generated a product ion at *m*/*z* 153 after the loss of hexose moiety [M−H−162]^−^ from the parent ion. Previously, Kakkar and Bais [38] reported the anti-microbial, antioxidant, anti-inflammatory, anti-cancer, anti-aging, anti-ulcer, anti-diabetic, and cardio-protective activities of protocatechuic acid. Punicalin (compound 7) is a hydrolyzable tannin found only in old man saltbush. 

##### Hydroxycinnamic Acids and Derivatives

Hydroxycinnamic acids are the most abundant class of phenolic acids in fruits, herbs, and medicinal plants. A total of 24 phenolic metabolites were identified as hydroxycinnamic acids and their derivatives in these selected Australian native plants. Cinnamic acid (compound 15), 3-caffeoylquinic acid (compound 16), Ferulic acid (compound 18), caffeic acid (compound 21), sinapic acid (compound 22), *p*-coumaric acid (compound 23), syringic acid (compound 27), rosmarinic acid (compound 28), chicoric acid (compound 30) were confirmed through external standards. Compound 8 (verbascoside A), compound 22 (sinapic acid), compound 26 (hydroxycaffeic acid), and compound 27 (syringic acid) were only tentatively identified in sandalwood nuts, while compound 10 (1,2,2′-triferuloylgentiobiose) and compound 14 (ferulic acid 4-*O*-glucuronide) were only identified in wattle seeds. Compounds 15, 18, 20, and 21 were only identified in lemongrass. Mainly, phenolic acids show the fragmentation pattern through the removal of CO_2_ (44 Da) and hexosyl moiety (162 Da) from the parent ions [9,13]. Compound 13 (3-sinapoylquinic acid) at ESI^−^ *m*/*z* 397.0927 produced fragment ions at *m*/*z* 223 (sinapic acid) and *m*/*z* 191 (quinic acid) and was tentatively identified in sandalwood nuts and wattle seeds. Compounds 14, 17, and 31 generated products ions at *m*/*z* 193 (ferulic acid), *m*/*z* 163 (coumaric acid), and *m*/*z* 179 (caffeic acid) after the removal of glucuronide moiety (176 Da) and hexosyl moiety (162 Da), respectively from their parent ions. Compounds 14, 17, and 31 were tentatively identified as ferulic acid 4-*O*-glucuronide, *p*-coumaric acid 4-*O*-glucoside, and caffeic acid 4-*O*-glucoside, respectively. Compounds 15, 21, 23, and 26 produced fragment ions at *m*/*z* 103, *m*/*z* 135, *m*/*z* 119, and *m*/*z* 151, respectively, after the loss of CO_2_ (44 Da) from their precursor ions. Compounds 15, 21, 23, and 26 were tentatively identified as cinnamic acid, caffeic acid, *p*-coumaric acid, and hydroxycaffeic acid, respectively. Compound 18 at ESI^−^ *m*/*z* 193.0499 produced fragment ions at *m*/*z* 178, *m*/*z* 149, and *m*/*z* 134 after the removal of [M−H−CH_3_], M−H−CO_2_], and [M−H−CH_3_+CO_2_], respectively from the parent ion. Compound 18 was tentatively identified as ferulic acid. Ferulic acid is well known due to its antioxidant, anti-diabetic, anti-cancer, anti-aging activity, radioprotective effect, pulmonary protective, neuro-protective effect, and hypotensive effect [39]. Compound 10 at ESI^−^ *m*/*z* 869.2495 generated product ions at *m*/*z* 693 (C_32_H_38_O_17_) and *m*/*z* 517 (C_22_H_30_O_14_) after the removal of one feruloyl unit and two feruloyl units, respectively from the precursor ion. Compound 10 was tentatively identified as 1,2,2′-triferuloylgentiobiose in wattle seeds. Previously, Passo Tsamo et al. [40] reported 1,2,2′-triferuloylgentiobiose in banana cultivars with the same MS/MS spectra. 

#### 3.4.2. Flavonoids

Flavonoids are the most abundant class of phenolic compounds. More than 10,000 flavonoids have been reported in nature [8]. We putatively identified a total of 62 flavonoids including 11 flavanols, 9 flavanones, 16 flavones 25 flavonols, and 4 chalcones and dihydrochalcones in selected Australian native plants (Table 3).

##### Flavanols

Flavanols or favan-3-ols are also called monomeric flavanols including catechins, epicatechin, gallocatechin, epigallocatechin, and their gallate derivatives. They are the most common flavonoids due to their diversity in chemical structures and biological functions. Compound 34 (procyanidin trimer C1) was tentatively identified in wattle seeds, which produced fragment ions at *m*/*z* 739, *m*/*z* 713, and *m*/*z* 695 in negative mode after the loss of heterocyclic ring fission [M−H−126], retro Diels–Alder [M−H−125], and loss of H_2_O (18 Da) from the latter product ion. Previously, Ali et al. [13] reported procyanidin trimer C1 in nutmeg and cinnamon. Compounds 35 and 36 at ESI^−^ *m*/*z* 441.0818 and 451.1236 generated a common product ion at *m*/*z* 289 which is a characteristic mass of catechin after the loss of gallate and hexose moiety, respectively, from their precursor ions. Therefore, compounds 35 and 36 were tentatively identified as (+)-catechin 3-*O*-gallate and catechin 3′-glucoside. Compound 42 at ESI^−^ *m*/*z* 289.0710 produced fragment ions at *m*/*z* 245, *m*/*z* 205, and *m*/*z* 179 after the loss of CO_2_ [M−H−44]^−^, flavonoid ring A [M−H−84]^−^ and flavonoid B ring [M−H−110]^−^, respectively from the parent ion. So, compound 42 was tentatively identified as (+)-catechin in sandalwood nuts and wattle seeds [41]. Compound 39 (procyanidin B2) produced fragment ions at *m*/*z* 451, 425, and 289 after the cleavage between the C4–C5 ring and O–C2 of one pyran ring, which caused the removal of phloroglucinol molecule (A-ring) from the parent ion (126 Da) and resulted in the product ions at *m*/*z* 451 and *m*/*z* 425 [41]. They are well-known for their antioxidant, anti-inflammatory, anti-cancer, and cardio-protective properties [42]. Catechins are the building blocks of condensed tannins commonly known as proanthocyanidins which have a wide range of pharmacological properties [43]. 

##### Flavones and Flavanones

In this context, a total of 24 flavonoids were putatively identified in these selected Australian native plants as flavones and flavanones (Table 3). Compounds 46, 61, 67, and 68 were only identified in lemongrass while compounds 57 and 60 were only identified in wattle seeds. Moreover, compound 52 was only identified in the old man saltbush. Compounds 47 and 48 at ESI^−^ *m*/*z* 477.1030 and *m*/*z* 741.2230 generated product ions at *m*/*z* 301 and *m*/*z* 579 after the loss of glucuronide [M−H−176] and hexose moiety [M−H−162], respectively from their precursor ions. Compound 49 (neoeriocitrin) was tentatively identified in lemongrass, sandalwood nuts, and wattle seeds, which produced fragment ions at *m*/*z* 459, 287, 151 after the removal of C_8_H_8_O_2_ [M−H−136]^−^, rhamnoside-glucoside moiety [M−H−308]^−^ and rhamnoside-glucoside moiety plus C_8_H_8_O_2_ [M−H−444]^−^, respectively, from the parent ion [44]. Previously, neoeriocitrin was identified in the exocarpium citri grandis extract [44], while compound 51 (naringin) generated product ions at m/z 459, 313, and 271 through the neutral loss of C_8_H_8_O (120 Da), C_8_H_8_O plus rhamnoside (266 Da) and rhamnoside plus glucoside (308 Da) from the parent ions, respectively. Naringin was tentatively identified in lemongrass, old man saltbush, and sandalwood nuts. Compounds 58 (swertisin) and 71 (diosmin) were identified through MS/MS spectra of pure standards.

##### Flavonols, Chalcones, and Dihydrochalcones

A total of 25 flavonols and 4 chalcones and dihydrochalcones were tentatively identified in selected Australian native plants. Compounds 72, 73, 79, 80, 85, 86, 87, 93, 94 and 96 product fragment ions at *m*/*z* 317, 301, 317, 315, 319, 287, 303, 285, 317 and 287, respectively after the loss of arabinoside (132 Da), rhamnoside (146 Da), glucoside (162 Da), glucuronide (176) and rutinoside (308 Da) from their precursor ions. Compounds 77, 80, 83, and 87 were only identified in old man saltbush while compounds 79 and 82 were only identified in sandalwood buts. Compounds 78, 88, 90, and 92 to 94 were only identified in lemongrass while compound 89 (quercetin 3-*O*-(6″-malonyl)-glucoside) was only identified in wattle seeds. Previously, quercetin 3-*O*-arabinoside and myricetin 3-*O*-rhamnoside were reported in mint and lemon [16]. Compound 75 (kaempferol 3,7-*O*-diglucoside) generated product ions at *m*/*z* 447 and 285 after the loss of one hexose moiety (162 Da) and two glucoside units (324 Da) from the precursor ion, respectively, while 3,7-dimethylquercetin (compound 87) produced fragment ions at m/z 316 and 301 after the loss of CH_3_ [M+H−CH_3_]^+^ and CO [M+H−CO]^+^, respectively, from the parent ion. Previously, 3,7-dimethylquercetin was reported in oregano, basil, sage, rosemary, and mint [9]. 

#### 3.4.3. Isoflavonoids

In isoflavonoids, ring A (phenyl ring) is fused with C-ring (six-membered heterocyclic ring) and another phenyl B-ring at the C3 position, while the B-ring is substituted to the C2 position in flavonoids [45]. These plants’ secondary metabolites contain a 3-phenylchroman skeleton which is biogenetically derived from the 2-phenylchroman (a basic skeleton of flavonoids) and more than 2400 isoflavonoids have been identified in plants [46]. It is the first time that we tentatively identified a total of 18 isoflavonoids in these selected Australian native plants. Compound 98 at ESI^−^ *m*/*z* 315.0866 was identified in old man saltbush and lemongrass, which produced fragment ions at *m*/*z* 300, *m*/*z* 285 and *m*/*z* 135 after the loss of [M−H−CH_3_], [M−H−2CH_3_] and [M−H−C_10_H_12_O_3_], respectively, from the parent ion. Compound 98 was tentatively characterized as violanone. Previously, Liu et al. [47] also reported violanone in the extract of *Dalbergia odorifera*. Compounds 95, 99, 102, 107, and 108 generated product ions at *m*/*z* 431, 241, 283, 253, and 269 after the loss of glucuronide moiety (176 Da) from their precursor ions, respectively. 

#### 3.4.4. Lignans and Stilbenes

Stilbenes are natural phytochemicals that contain a 1,2-diphenylethylene (a basic skeleton of stilbenoids) and have various pharmacological properties including antioxidant, antimicrobial, anti-cancer, anti-inflammatory, anti-diabetic, anti-aging, cardio-protective, and neuro-protective properties [48]. In this study, a total of five stilbenes were tentatively identified in selected Australian native plants (Table 3). Compound 121 at ESI^−^ *m*/*z* 243.0679 produced fragment ions at *m*/*z* 225 and *m*/*z* 201 after the loss of a water molecule [M−H−H_2_O]^−^ and carbon dioxide [M−H−CO_2_]^−^, respectively, from the parent ion. Compound 121 was tentatively identified as a piceatannol and was only identified in wattle seeds. Previously, it was identified in dill leaves and fenugreek [9]. 

Lignans are a subgroup of non-flavonoid phenolic compounds which comprised two phenylpropane units (C6–C3). In this study, a total of 11 lignans were tentatively identified in selected Australian native plants (Table 3). Compounds 124 and 134 were only identified in wattle seeds and sandalwood nuts, respectively. Todolactol A (compound 124) at ESI^−^ *m*/*z* 375.1443 was only identified in wattle seeds while compound 134 (7-hydroxysecoisolariciresinol) at ESI^−^ *m*/*z* 373.2017 was only identified in sandalwood nuts. Compounds 126 (7-oxomatairesinol), 127 (conidendrin), and 131 (schisandrin) were only identified in the old man saltbush, while compounds 128 (sesaminol 2-*O*-triglucoside) and 130 (1-acetoxypinoresinol) were only detected in lemongrass. Stilbenes and lignans are widely distributed in plants and have beneficial health properties. 

#### 3.4.5. Other Polyphenols

In this context, a total of 21 other polyphenols including 6 coumarins and derivatives, phenolic terpenes (3), tyrosols (4), hydroxybenzoketones (1), hydroxyphenylpropenes (1), cyclitol (1), and other polyphenols (5) were putatively identified in selected Australian native plants (Table 3). Umbelliferone (compound 137) was tentatively identified in lemongrass only at ESI^−^ *m*/*z* 161.0246, which generated two product ions at *m*/*z* 133 and *m*/*z* 117 after the loss of CO (28 Da) and CO_2_ (44 Da) from the precursor ion, respectively. Compounds 144, 145, and 146 generated product ions at *m*/*z* 287, 105, and 301 after the loss of CO_2_ [M−H−44]^−^ from their precursor ions, respectively. Compounds 144, 145, and 146 were tentatively identified as carnosic acid, carvacrol, and rosmanol, respectively. These compounds are phenolic terpenes which have been reported for their antioxidant activity [49]. Pyrogallol (compound 151) was identified in lemongrass, wattle seeds, and old man saltbush which produced fragment ions at *m*/*z* 107 and 97 after the loss of a water molecule (18 Da) and CO (28 Da), respectively from the precursor ion. Previously, pyrogallol was confirmed through LC-QTOF-MS/MS and NMR by Zhao et al. [50]. 

### 3.5. Distribution of Phenolic Metabolites in Australian Native Plants

**Figure 3:**
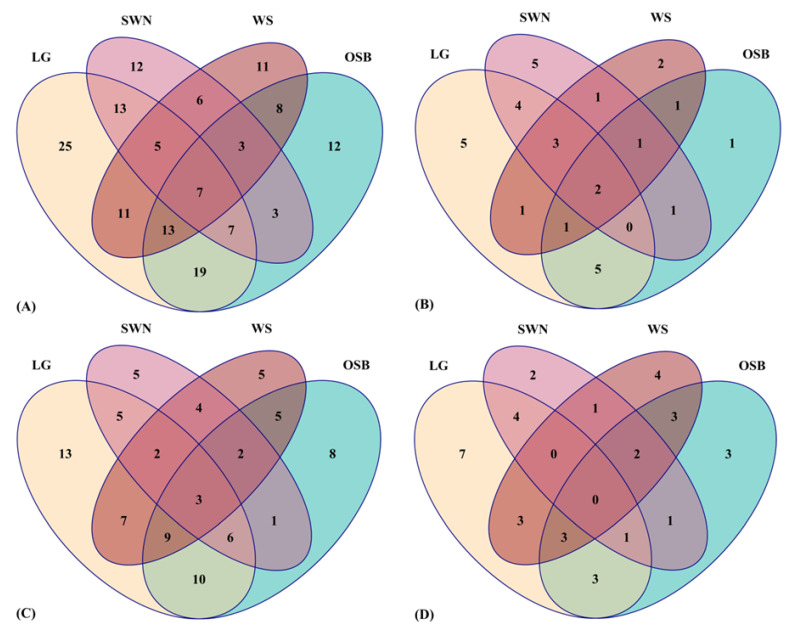
Distribution of phenolic compounds in Australian native plants. (**A**) a total number of phenolic metabolites, (**B**) a total number of phenolic acids, (**C**) a total number of flavonoids, (**D**) total other phenolic metabolites in Australian native lemongrass (LG), sandalwood nuts (SWN), wattle seeds (WS), and old man saltbush (OSB).

The distribution of phenolic metabolites in Australian native lemongrass, old man saltbush, wattle seeds, and sandalwood nuts was achieved statistically by using a Venn diagram in R studio, given in Figure 3.

The Venn diagram (Figure 3A) indicates that a total of 25 (16%) unique phenolic compounds were identified in native lemongrass, while a total of 11 (7%), 12 (8%), and 12 (8%) unique phenolic metabolites were identified in wattle seeds, sandalwood nuts, and old man saltbush, respectively. This indicates that lemongrass has a more diverse range of phenolic metabolites that may contribute to its higher TPC, TFC, and antioxidant potential compared to other plant extracts (Table 1, Figure 1). A Venn diagram (Figure 3B) depicts the total phenolic acids in selected Australian native plants. It was observed that lemongrass and sandalwood nuts have a greater variety (15.2%) of unique phenolic acids (5) as compared to old man saltbush and wattle seeds, in which only one (3.0%) and two (6.0%) unique phenolic acids were observed. This diagram further depicts that four (12.1%) of phenolic acids in both lemongrass and sandalwood nuts were similar while only two (6.0%) phenolic acids overlapped in all four plants. Figure 3C represents the total number of flavonoids in Australian native plants. It shows that the highest number of unique flavonoids, 13 (15.4%), was observed in lemongrass while the lowest number of unique flavonoids, 5 (6.0%), was in wattle seeds and sandalwood nuts. Moreover, sandalwood nuts contain a total of 8 (9.5%) unique flavonoids. A total of 10 (11.9%) flavonoids were overlapped in lemongrass and old man saltbush while a total of 5 (6.0%) were overlapped in lemongrass and wattle seeds, and wattle seeds and old man saltbush. A total of 3 (3.6%) flavonoids overlapped in all four selected plants. Figure 3D shows the total number of other phenolic metabolites in Australian native lemongrass, wattle seeds, sandalwood nuts, and old man saltbush. The highest numbers of unique other phenolic metabolites 7 (18.9%) were observed in lemongrass while the lowest numbers of unique phenolic metabolites 2 (5.4%) were observed in sandalwood nuts. Interestingly, a total of three (8.1%) other phenolic metabolites overlapped in lemongrass and wattle seeds, lemongrass and old man saltbush, wattle seeds and old man saltbush, and lemongrass, old man saltbush, and wattle seeds. It was observed that none of the other phenolic metabolites overlapped in all four plants. The Venn diagram is a useful, powerful, and versatile tool that can quickly analyze a large set of data and converts it into simple and digestible information.

### 3.6. Heatmap Hierarchical Clustering of Quantified Phenolic Metabolites

**Figure 4:**
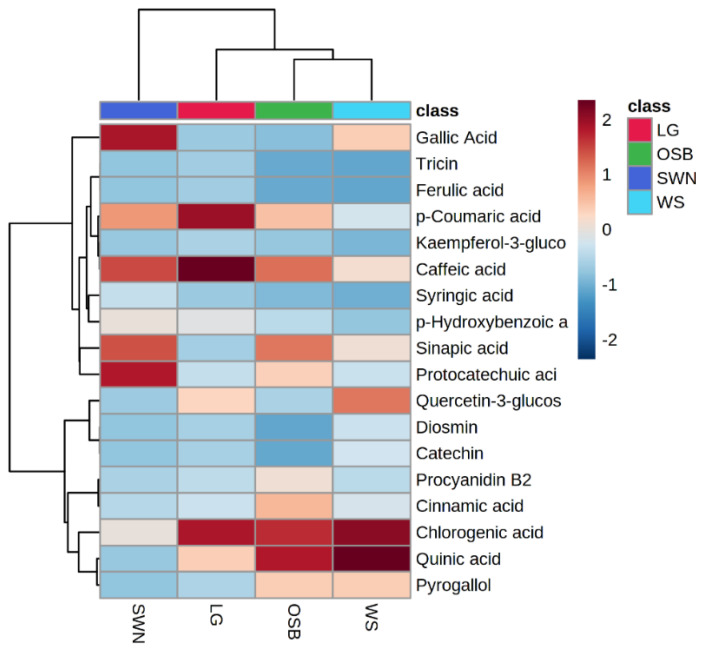
Heatmap hierarchical clustering of quantified phenolic metabolites.

In this study, we quantified/semi-quantified a total of 18 phenolic metabolites in these selected Australian native plants (Table S2). The highest numbers of phenolic metabolites (15) were quantified in lemongrass while only three phenolic metabolites were quantified in old man saltbush. The highest concentration of caffeic acid (445.21 ± 32.77 μg/g), *p*-coumaric acid (393.32 ± 39.56 μg/g), chlorogenic acid (377.65 ± 4.26 μg/g), and quercetin-3-glucoside (151.35 ± 11.34 μg/g) were measured in lemongrass, while the lowest concentration of diosmin (11.04 ± 2.14 μg/g) and catechin (11.54 ± 3.07 μg/g) were quantified in wattle seeds. Chlorogenic acid (18.76 ± 6.34 μg/g), quinic acid (19.64 ± 3.92 μg/g), and pyrogallol (11.02 ± 1.63 μg/g) were also quantified in old man saltbush. Ferulic acid (12.17 ± 3.11 μg/g), tricin (12.34 ± 2.31 μg/g), kaempferol-3-glucoside (21.45 ± 4.12 μg/g), procyanidin B2 (46.75 ± 6.56 μg/g), and cinnamic acid (61.30 ± 17.31 μg/g) were only quantified in lemongrass, while sinapic acid (77.17 ± 6.85 μg/g) and syringic acid (17.04 ± 3.45 μg/g) were only quantified in sandalwood nuts. The highest concentration of gallic acid (93.32 ± 18.44 μg/g) was quantified in sandalwood nuts while the lowest concentration of gallic acid (18.53 ± 6.15 μg/g) was measured in wattle seeds. Heatmap hierarchical clustering was also conducted for quantified phenolic metabolites through MetaboAnalyst (www.metaboanalyst.ca) accessed on 28 August 2022 given in Figure 4. It indicates that two column-wise and eight row-wise clusters were generated in quantified phenolic metabolites of lemongrass, old man saltbush, sandalwood nuts, and wattle seeds. The dense red color indicates higher concentration, while the blue color depicts lower or zero concentration in selected Australian native fruits. Lemongrass contained the highest concentration of caffeic acid, *p*-coumaric acid, and chlorogenic acid, while sandalwood nuts had the highest concentration of gallic acid, caffeic acid, protocatechuic acid, and sinapic acid. Wattle seeds quantified a higher concentration of chlorogenic acid and quinic acid than other phenolic metabolites. 

## 4. Conclusions

These data indicate that the selected Australian native plants contained a diverse range of phenolic metabolites. A total of 155 phenolic metabolites (100 in lemongrass, 56 in sandalwood nuts, 64 in wattle seeds, and 70 in old man saltbush) were tentatively identified. Phenolic metabolites have significant health potential; therefore, these plants could be utilized in the pharmaceutical, medicinal, and food industries. Chlorogenic acid, *p*-coumaric acid, caffeic acid, protocatechuic acid, quinic acid, sinapic acid, gallic acid, quercetin 3-glucoside, pyrogallol, and cinnamic acid are abundant phenolic metabolites in selected Australian native plants. The significant antioxidant potential and in-depth phytochemical composition of these selected Australian native plants will further explore the use of these plants in medicinal, cosmetic, food, and feed industries.

## Figures and Tables

**Table 1:** Total polyphenol contents of Australian native plants.

Variables	TPC (mg GAE/g)	TFC (mg QE/g)	TCT (mg CE/g)
Sandalwood nuts	8.54 ± 0.33 ^b^	2.81 ± 0.21 ^b^	1.12 ± 0.06 ^cd^
Lemongrass	15.09 ± 0.88 ^a^	3.07 ± 0.08 ^a^	1.36 ± 0.08 ^c^
Old man saltbush	6.39 ± 0.25 ^c^	2.32 ± 0.12 ^b^	1.99 ± 0.02 ^b^
Wattle seeds	4.17 ± 0.33 ^d^	0.67 ± 0.05 ^c^	2.88 ± 0.10 ^a^

Total Phenolic Content (TPC), Total Flavonoid Content (TFC), Total Condensed Tannins (TCT), and Values (mean ± standard deviation; *n* = 3) within the column are significantly different (*p* < 0.05) from each other represented by the superscript letters (^a–d^).

**Table 2:** Pearson correlation of phenolic contents and antioxidant activities in Australian native plants.

Variables	TPC	TFC	TCT	DPPH	ABTS	FRAP	RPA	PMA	FICA
TFC	0.80								
TCT	−0.72	**−0.96**							
DPPH	**0.98**	0.68	−0.57						
ABTS	0.64	0.31	−0.06	0.76					
FRAP	0.65	0.81	**−0.94**	0.49	−0.15				
RPA	**0.99**	0.78	−0.74	**0.96**	0.54	0.71			
PMA	0.55	0.70	−0.88	0.38	−0.29	**0.99**	0.63		
FICA	**0.97**	0.64	−0.52	1.00	0.78	0.45	**0.94**	0.34	
^•^OH-RSA	**0.98**	0.80	−0.67	0.98	0.75	0.54	**0.94**	0.41	**0.97**

Values in bold are different from 0 with a significance level alpha = 0.1.

**Table 3:** LC-ESI-QTOF-MS/MS Identification of Phenolic Metabolites from Australian Native Plants.

No.	Proposed Compounds	Molecular Formula	RT (min)	Mode of Ionization	Theoretical (*m*/*z*)	Observed (*m*/*z*)	Mass Error (ppm)	MS/MS	Samples
	**Phenolic acids**								
**Hydroxybenzoic acid**									
1	* Protocatechuic acid	C_7_H_6_O_4_	6.821	[M−H]^−^	153.0193	153.0186	−4.6	109	LG, SWN
2	* Gallic acid	C_7_H_6_O_5_	6.913	[M−H]^−^	169.0142	169.0134	4.6	125	WS, SWN
3	Protocatechuic acid 4-*O*-glucoside	C_13_H_16_O_9_	10.169	[M−H]^−^	315.0721	315.0735	4.6	153, 109	LG
4	* p-Hydroxybenzoic acid	C_7_H_6_O_3_	16.180	[M−H]^−^	137.0244	137.0240	−2.9	93	LG, SWN
5	* Vanillic acid	C_8_H_8_O_4_	17.114	[M−H]^−^	167.0350	167.0345	−3.0	152, 123, 108	LG, SWN, WS
6	* Ellagic acid	C_14_H_6_O_8_	25.216	** [M−H]^−^	300.9990	300.9988	−0.7	284, 257	SWN, OSB
7	Punicalin	C_34_H_22_O_22_	48.689	[M+H]+	783.0676	783.0646	−3.8	765, 737, 675, 617, 169	OSB
**Hydroxycinnamic acids**									
8	Verbascoside A	C_31_H_40_O_16_	3.866	[M−H]^−^	667.2243	667.2268	3.7	283, 94	SWN
9	1-Feruloyl-5-caffeoylquinic acid	C_26_H_26_O_12_	3.943	[M−H]−	529.1351	529.1343	−1.5	193, 191, 179, 135	OSB, LG
10	1,2,2′-Triferuloylgentiobiose	C_42_H_46_O_20_	3.951	[M−H]−	869.2509	869.2495	−1.6	693, 517	WS
11	1-Sinapoyl-2-feruloylgentiobiose	C_33_H_40_O_18_	3.951	[M−H]−	723.2142	723.2149	1.0	529, 499	WS, OSB
12	3-*p*-Coumaroylquinic acid	C_16_H_18_O_8_	4.271	[M−H]−	337.0929	337.0927	−0.6	191, 119	WS, LG, OSB, SWN
13	3-Sinapoylquinic acid	C_18_H_22_O_10_	4.366	[M−H]−	397.1140	397.1116	−6.0	223, 191	SWN, WS
14	Ferulic acid 4-*O*-glucuronide	C_16_H_18_O_10_	6.634	[M−H]−	369.0827	369.0847	5.4	193	WS
15	* Cinnamic acid	C_9_H_8_O_2_	7.678	[M−H]−	147.0451	147.0446	−2.2	103	LG
16	* 3-Caffeoylquinic acid	C_16_H_18_O_9_	13.294	** [M−H]−	353.0878	353.0874	−1.1	191, 179, 161	SWN, WS, LG, OSB
17	*p*-Coumaric acid 4-*O*-glucoside	C_15_H_18_O_8_	14.524	** [M−H]−	325.0929	325.0920	−2.8	163	LG, OSB
18	* Ferulic acid	C_10_H_10_O_4_	15.335	[M−H]−	193.0506	193.0499	−3.6	178, 149, 134	LG
19	3-Feruloylquinic acid	C_17_H_20_O_9_	15.335	[M−H]−	367.1034	367.1038	1.1	193, 191, 134	LG, SWN, WS
20	1-*O*-Sinapoyl-ꞵ-D-glucose	C_17_H_22_O_10_	16.295	[M−H]−	385.1140	385.1145	1.3	223, 193	LG
21	* Caffeic acid	C_9_H_8_O_4_	18.553	[M−H]−	179.0350	179.0358	2.9	161, 135	LG
22	* Sinapic acid	C_11_H_12_O_5_	22.223	[M−H]−	223.0612	223.0606	−2.7	193, 179, 149, 134	SWN
23	* *p*-Coumaric acid	C_9_H_8_O_3_	24.583	[M−H]−	163.0400	163.0395	−3.1	119	SWN, LG
24	1,5-Dicaffeoylquinic acid	C_25_H_24_O_12_	24.731	** [M−H]−	515.1195	515.1204	2.6	191, 179, 135	LG, OSB
25	Verbascoside	C_29_H_36_O_15_	26.408	** [M−H]−	623.1981	623.1985	0.6	462, 461, 161	OSB, LG
26	Hydroxycaffeic acid	C_9_H_8_O_5_	26.690	[M−H]−	195.0299	195.0299	0.0	177, 151	SWN
27	* Syringic Acid	C_9_H_10_O_5_	28.680	[M−H]−	197.0455	197.0455	0.0	182, 153, 138, 121	SWN
28	* Rosmarinic acid	C_18_H_16_O_8_	29.245	[M−H]−	359.0772	359.0781	2.5	197, 179, 161, 135	OSB, SWN, LG
29	1,2-Diferuloylgentiobiose	C_32_H_38_O_17_	29.283	** [M−H]−	693.2036	693.2055	2.7	193, 134	LG, OSB
30	Chicoric acid	C_22_H_18_O_12_	37.085	** [M+H]+	475.0871	475.0873	0.4	293, 311	LG, WS
31	Caffeic acid 4-*O*-glucoside	C_15_H_18_O_9_	45.661	[M+H]+	343.1024	343.1016	−2.3	179	WS, LG
	Hydroxyphenylacetic acids								
32	2-Hydroxy-2-phenylacetic acid	C_8_H_8_O_3_	14.301	[M−H]−	151.0400	151.0400	0.0	136, 92	LG, SWN
33	Homovanillic acid	C_9_H_10_O_4_	32.463	[M−H]−	181.0506	181.0497	−5.0	163	SWN
	**Flavonoids**								
**Flavanols**									
34	Procyanidin trimer C1	C_45_H_38_O_18_	3.871	[M−H]−	865.1985	865.2049	7.4	739, 713, 695, 577, 451	WS
35	(+)-Catechin 3-*O*-gallate	C_22_H_18_O_10_	3.940	** [M−H]−	441.0827	441.0818	−2.0	289	SWN, WS, LG, OSB
36	Catechin 3′-glucoside	C_21_H_24_O_11_	3.977	[M−H]−	451.1246	451.1236	−2.2	289	WS, SWN, OSB, LG
37	4′-*O*-Methylepigallocatechin	C_16_H_16_O_7_	4.527	[M−H]−	319.0823	319.0804	−6.0	289, 245	WS
38	4″-*O*-Methylepigallocatechin 3-*O*-gallate	C_23_H_20_O_11_	9.014	** [M−H]−	471.0933	471.0927	−1.3	305, 183, 139	LG, OSB, WS
39	* Procyanidin B2	C_30_H_26_O_12_	18.412	** [M−H]^−^	577.1351	577.1366	2.6	451, 425, 407, 289	WS, LG
40	Epigallocatechin 3-*O*-gallate-7-*O*-glucoside-4″-*O*-glucuronide	C_34_H_36_O_22_	18.553	** [M−H]−	795.1625	795.1625	0.0	305, 289, 245	LG, WS, OSB
41	4′,4″-Dimethylepigallocatechin gallate	C_24_H_22_O_11_	19.568	[M−H]^−^	485.1089	485.1106	3.5	305, 289	LG, WS
42	* (+)-Catechin	C_15_H_14_O_6_	41.850	** [M−H]^−^	289.0717	289.0710	−2.4	245, 205, 179	SWN, WS
43	4′-*O*-Methyl-(-)-epicatechin 3′-*O*-glucuronide	C_22_H_24_O_12_	45.631	[M−H]−	479.1195	479.1208	2.7	461, 435, 303	LG, SWN
44	(-)-Epigallocatechin 3′-*O*-glucuronide	C_21_H_22_O_13_	57.250	** [M+H]^+^	483.1133	483.1142	1.9	307	OSB, WS, LG
45	(+)-Gallocatechin	C_15_H_14_O_7_	68.325	[M+H]^+^	307.0813	307.0803	−3.3	291	OSB
	**Flavanones**								
46	Hesperidin	C_28_H_34_O_15_	4.210	[M−H]−	609.1825	609.1821	−0.7	301	LG
47	Hesperetin 3′-*O*-glucuronide	C_22_H_22_O_12_	4.828	[M−H]−	477.1038	477.1030	−1.7	301	SWN, WS
48	Narirutin 4′-*O*-glucoside	C_33_H_42_O_19_	4.924	[M−H]−	741.2247	741.2230	−2.3	579	LG, OSB, WS
49	Neoeriocitrin	C_27_H_32_O_15_	15.335	[M−H]−	595.1668	595.1655	−2.2	459, 287, 151	LG, SWN, WS
50	Didymin	C_28_H_34_O_14_	25.479	[M−H]−	593.1876	593.1880	0.7	447, 285, 151	SWN, WS, LG, OSB
51	Naringin	C_27_H_32_O_14_	28.576	[M−H]−	579.1719	579.1699	−3.5	459, 313, 271	LG, OSB, SWN
52	Naringin 6′-malonate	C_30_H_34_O_17_	38.465	[M−H]−	665.1723	665.1711	−1.8	579	OSB
53	Naringenin	C_15_H_12_O_5_	44.409	[M−H]−	271.0612	271.0623	4.1	151, 119	SWN
54	Naringenin 7-*O*-glucoside	C_21_H_22_O_10_	49.237	[M−H]−	433.1140	433.1140	0.0	271	LG, OSB, WS
55	Hesperetin 5,7-*O*-diglucuronide	C_28_H_30_O_18_	60.331	** [M+H]+	655.1505	655.1518	2.0	303	WS, OSB
**Flavones**									
56	Tetramethylscutellarein	C_19_H_18_O_6_	4.050	** [M−H]−	341.1030	341.1030	0.0	341	LG, OSB, WS
57	Syringetin-3-*O*-glucoside	C_23_H_24_O_13_	7.166	[M−H]−	507.1144	507.1165	4.1	345	LG, OSB, SWN
58	* Swertisin	C_22_H_22_O_10_	10.402	[M−H]−	445.1140	445.1171	7.0	325, 297, 282	WS
59	Apigenin 6,8-C-arabinoside-C-glucoside	C_26_H_28_O_14_	18.932	[M−H]−	563.1406	563.1402	−0.7	269	LG, OSB, WS
60	6-Hydroxyluteolin 7-*O*-rhamnoside	C_21_H_20_O_11_	18.932	** [M−H]−	447.0933	447.0931	−0.4	429, 301, 163	LG, WS
61	Chrysoeriol 7-*O*-glucoside	C_22_H_22_O_11_	21.244	[M−H]−	461.1089	461.1101	2.6	289	WS
62	Rhoifolin	C_27_H_30_O_14_	22.656	[M−H]−	577.1563	577.1615	9.0	431, 269	LG
63	Tricin 7-neohesperidoside	C_29_H_34_O_16_	22.886	[M−H]−	637.1774	637.1785	1.7	329	OSB, WS
64	8-Methoxyluteolin	C_16_H_12_O_7_	25.360	[M−H]^−^	315.0510	315.0513	1.0	300	SWN, WS
65	3,4′,7-Tetrahydroxyflavone	C_15_H_10_O_6_	26.486	[M−H]−	285.0404	285.0404	0.0		SWN, LG
66	Apigenin 6,8-di-C-glucoside	C_27_H_30_O_15_	27.921	** [M−H]^−^	593.1512	593.1517	0.8	269	LG, OSB
67	6-Hydroxyluteolin	C_15_H_10_O_7_	28.680	** [M−H]^−^	301.0353	301.0348	−1.7	285	SWN, LG
68	6-Hydroxyflavone	C_15_H_10_O_3_	36.774	[M−H]^−^	237.0557	237.0568	4.6	237	LG
69	Lonicerjaponin B	C_34_H_44_O_17_	41.301	[M−H]^−^	723.2505	723.2499	−0.8	723	LG
70	Cirsilineol	C_18_H_16_O_7_	65.460	[M+H]^+^	345.0969	345.0956	−3.8	303, 312, 297, 284	LG, OSB, WS
71	* Diosmin	C_28_H_32_O_15_	69.786	** [M+H]^+^	609.1814	609.1783	−5.1	301	WS, LG
**Flavonols**									
72	Myricetin 3-*O*-arabinoside	C_20_H_18_O_12_	4.950	** [M−H]−	449.0725	449.0736	2.4	317	LG, OSB
73	Quercetin 3-*O*-arabinoside	C_20_H_18_O_11_	5.145	** [M−H]−	433.0776	433.0792	3.7	301	SWN, OSB, WS
74	3-Methoxysinensetin	C_21_H_22_O_8_	12.608	[M−H]−	401.1242	401.1241	−0.2	327, 209	OSB, SWN, LG
75	Kaempferol 3,7-*O*-diglucoside	C_27_H_30_O_16_	14.751	[M−H]−	609.1461	609.1464	0.5	447, 285	LG, SWN
76	Kaempferol 3-*O*-xylosyl-glucoside	C_26_H_28_O_15_	16.783	[M−H]−	579.1355	579.1353	−0.3	285	LG, OSB
77	3-Methoxynobiletin	C_22_H_24_O_9_	17.998	[M−H]−	431.1347	431.1357	2.3	401, 387	OSB
78	Kaempferide	C_16_H_11_O_6_	18.000	[M−H]−	298.0483	298.0498	5.0	283, 151	LG
79	Myricetin 3-*O*-rhamnoside	C_21_H_20_O_12_	24.447	[M−H]−	463.0882	463.0896	3.0	317	SWN
80	Isorhamnetin 3-*O*-rutinoside	C_28_H_32_O_16_	24.961	** [M−H]−	623.1617	623.1667	8.0	315	OSB
81	Spinacetin 3-*O*-glucosyl-(1->6)-glucoside	C_29_H_34_O_18_	28.842	[M−H]−	669.1672	669.1671	−0.1	669	OSB, SWN, LG
82	6,8-Dihydroxykaempferol	C_15_H_10_O_8_	33.805	[M−H]−	317.0303	317.0314	3.5	285	SWN
83	Taxifolin 4′,7-diglucoside	C_27_H_32_O_17_	35.838	** [M−H]−	627.1567	627.1542	−4.0	303	OSB
84	Kaempferol 3,7,4′-*O*-triglucoside	C_33_H_40_O_21_	35.986	** [M−H]−	771.1989	771.1989	0.0	285	LG, OSB
85	Myricetin 3-*O*-glucoside	C_21_H_20_O_13_	50.735	** [M+H]+	481.0977	481.0980	0.6	319	OSB, LG
86	Kaempferol 3-*O*-rhamnoside	C_21_H_19_O_10_	52.448	** [M+H]+	432.1051	432.1037	−3.2	287	OSB, LG
87	Quercetin 4′-*O*-glucuronide	C_21_H_18_O_13_	54.978	[M+H]+	479.0820	479.0814	−1.3	303	OSB
87	3,7-Dimethylquercetin	C_17_H_14_O_7_	44.677	** [M+H]+	331.0813	331.0804	−2.7	316, 301	LG, OSB, SWN
88	* Tricin	C_17_H_14_O_7_	44.677	** [M−H]−	331.0813	331.0804	−2.7	316, 301	LG
89	Quercetin 3-*O*-(6″-malonyl)-glucoside	C_24_H_22_O_15_	64.747	** [M−H]−	549.0886	549.0900	2.5	301	WS
90	Quercetin 3-*O*-xylosyl-glucuronide	C_26_H_26_O_17_	64.800	[M+H]+	611.1243	611.1224	−3.1	303	LG
91	(-)-Epicatechin-epicatechin-galactoside	C_36_H_34_O_15_	66.810	** [M+H]+	707.1971	707.2000	4.1	291	WS, LG
92	Quercetin 3-*O*-glucosyl-xyloside	C_26_H_28_O_16_	66.838	[M+H]+	597.1450	597.1469	3.2	303	LG
93	Kaempferol 3-*O*-glucoside	C_21_H_20_O_11_	23.730	[M−H]−	447.093261	447.0947	3.2	285	LG
94	Isorhamnetin 3-*O*-glucuronide	C_22_H_20_O_13_	68.303	[M+H]+	493.0977	493.0994	3.4	317	LG
95	Quercetin 3-*O*-xylosyl-rutinoside	C_32_H_38_O_20_	68.764	** [M+H]+	743.2029	743.2019	−1.3	303	WS, SWN, OSB
96	Kaempferol 3-*O*-glucuronide	C_21_H_18_O_12_	69.376	[M+H]+	463.0871	463.0880	1.9	287	WS, OSB
**Isoflavonoids**									
97	Daidzin 4′-*O*-glucuronide	C_27_H_28_O_15_	3.075	** [M+H]^+^	593.1501	593.1491	−1.7	431	OSB, LG
98	6″-*O*-Malonyldaidzin	C_24_H_22_O_12_	6.634	** [M−H]−	501.1038	501.1015	−4.6	253	WS, LG
99	3′-*O*-Methylviolanone	C_18_H_18_O_6_	11.024	[M−H]−	329.1030	329.1025	−1.5	285, 163	OSB
100	Violanone	C_17_H_16_O_6_	11.105	* [M−H]−	315.0874	315.0867	−2.2	300, 285, 135	OSB, LG
101	Equol 7-*O*-glucuronide	C_21_H_22_O_9_	12.021	[M−H]−	417.1191	417.1198	1.7	241	OSB, SWN
102	Dihydrobiochanin A	C_16_H_14_O_5_	12.216	[M−H]−	285.0768	285.0767	−0.4	203, 175	OSB, WS
103	Sativanone	C_17_H_16_O_5_	12.341	** [M−H]−	299.0925	299.0933	2.7	284, 269, 225	OSB
104	Glycitein 4′-*O*-glucuronide	C_22_H_20_O_11_	15.551	** [M−H]−	459.0933	459.0918	−3.3	441, 283, 267	WS, OSB, LG
105	6″-*O*-Acetyldaidzin	C_23_H_22_O_10_	16.735	** [M−H]−	457.1140	457.1155	3.3	439, 253	LG, SWN, WS
106	Genistein 4′,7-*O*-diglucuronide	C_27_H_26_O_17_	18.356	** [M−H]−	621.1097	621.1073	−3.9	445, 427, 269	LG, OSB
107	3′-Hydroxymelanettin	C_16_H_12_O_6_	18.519	[M−H]−	299.0561	299.0556	−1.7	284	LG
108	6″-*O*-Acetylgenistin	C_23_H_22_O_11_	18.553	** [M−H]−	473.1089	473.1120	6.6	269, 59	LG
109	Daidzein 7-*O*-glucuronide	C_21_H_18_O_10_	19.221	[M−H]−	429.0827	429.0855	6.5	253	LG, WS
110	Formononetin 7-*O*-glucuronide	C_22_H_20_O_10_	30.443	[M−H]−	443.0983	443.0994	2.5	269	OSB, LG
111	3′,4′,5,7-Tetrahydroxyisoflavanone	C_15_H_12_O_6_	33.384	[M−H]−	287.0561	287.0547	−4.9	269, 179	SWN
112	3′-Hydroxy-*O*-desmethylangolensin	C_15_H_14_O_5_	43.831	[M−H]−	273.0768	273.0769	0.4	273	SWN
113	6″-*O*-Malonylglycitin	C_25_H_24_O_13_	44.248	[M−H]−	531.1144	531.1160	3.0	283, 267	LG
114	6″-*O*-Malonylgenistin	C_24_H_22_O_13_	44.567	** [M+H]^+^	519.1133	519.1126	−1.3	271	WS, OSB, WS
**Chalcones and Dihydrochalcones**									
115	Phloridzin	C_21_H_24_O_10_	4.309	[M−H]−	435.1297	435.1305	1.8		WS, SWN
116	Phloretin 2′-*O*-xylosyl-glucoside	C_26_H_32_O_14_	17.703	** [M−H]−	567.1719	567.1726	1.2	273	LG, SWN
117	Phloretin 2′-*O*-glucuronide	C_21_H_22_O_11_	23.564	[M−H]−	449.1089	449.1073	−3.6	273, 149	LG, OSB, SWN
118	Xanthohumol	C_21_H_22_O_5_	26.055	[M−H]−	353.1394	353.1392	−0.6	233, 119	LG
	**Stilbenes**								
119	Dihydroresveratrol	C_14_H_14_O_3_	4.160	[M−H]−	229.0870	229.0880	4.4	229	WS
120	Piceatannol 3-*O*-glucoside	C_20_H_22_O_9_	4.428	[M−H]−	405.1191	405.1161	−7.4	243	WS
121	Piceatannol	C_14_H_12_O_4_	4.674	[M−H]−	243.0663	243.0679	6.6	225, 201	WS
122	trans-Resveratrol 3-*O*-glucuronide	C_20_H_20_O_9_	10.023	[M−H]−	403.1034	403.1041	1.7	227	OSB, WS
123	Resveratrol 3-*O*-glucoside	C_20_H_22_O_8_	23.802	[M−H]−	389.1242	389.1248	1.5	227	LG, SWN
	**Lignans**								
124	Todolactol A	C_20_H_24_O_7_	5.816	[M−H]−	375.1449	375.1443	−1.6	357, 329	WS
125	Sesamin	C_20_H_18_O_6_	13.905	[M−H]−	353.1030	353.1015	−4.2	338, 163	OSB, SWN, LG
126	7-Oxomatairesinol	C_20_H_20_O_7_	15.308	[M−H]−	371.1136	371.1127	−2.4	358, 343, 328	OSB
127	Conidendrin	C_20_H_20_O_6_	16.050	[M−H]−	355.1187	355.1175	−3.4	337, 311, 309, 295	OSB
128	Sesaminol 2-*O*-triglucoside	C_36_H_46_O_22_	17.812	[M−H]−	829.2408	829.2448	4.8	369	LG
129	Trachelogenin	C_21_H_24_O_7_	21.905	[M−H]−	387.1449	387.1464	3.9	343, 329, 137	OSB, LG
130	1-Acetoxypinoresinol	C_22_H_24_O_8_	21.957	[M−H]−	415.1398	415.1394	−1.0	357	LG
131	Schisandrin	C_24_H_32_O_7_	28.132	[M−H]−	431.2075	431.2064	−2.6	431	OSB
132	Deoxyschisandrin	C_24_H_32_O_6_	51.605	[M−H]−	415.2126	415.2126	0.0	402, 347, 361, 301	SWN, OSB
133	Schisandrin C	C_22_H_24_O_6_	61.129	[M−H]−	383.1500	383.1505	1.3	367, 339, 311	WS, LG
134	7-Hydroxysecoisolariciresinol	C_22_H_30_O_5_	66.881	[M−H]−	373.2020	373.2017	−0.8	357, 355, 327, 221	SWN
	**Other polyphenols**								
**Coumarins and derivatives**									
135	Bergapten	C_12_H_8_O_4_	6.562	** [M−H]^−^	215.0350	215.0353	1.4	171	WS, OSB
136	Scopoletin	C_10_H_8_O_4_	14.304	[M−H]−	191.0350	191.0340	−5.2	175, 147	SWN, WS
137	Umbelliferone	C_9_H_6_O_3_	17.236	[M−H]−	161.0244	161.0246	1.2	133, 117	LG
138	Esculetin	C_9_H_6_O_4_	18.068	[M−H]−	177.0193	177.0205	6.8	133, 105	LG, SWN
139	Esculin	C_15_H_16_O_9_	19.490	[M−H]−	339.0721	339.0731	2.9	177	LG
140	Isopimpinellin	C_13_H_10_O_5_	26.790	[M−H]−	245.0455	245.0456	0.4	215, 201	SWN, OSB, WS
**Cyclitol**									
141	Quinic Acid	C_7_H_12_O_6_	15.086	[M−H]^−^	191.0561	191.0551	−5.2	173, 127, 85	LG, SWN
**Hydroxybenzoketones**									
142	Norathyriol	C_13_H_8_O_6_	3.909	[M−H]−	259.0248	259.0255	2.7	241, 231	OSB, SWN, WS
	Hydroxyphenylpropenes								
143	[6]-Gingerol	C_17_H_32_O_4_	65.884	[M−H]−	299.2228	299.2220	−2.7	299	WS, OSB, LG
**Phenolic terpenes**									
144	Carnosic acid	C_20_H_28_O_4_	59.906	[M−H]−	331.1915	331.1930	4.5	287	OSB, LG
145	Carvacrol	C_10_H_14_O	66.059	[M−H]−	149.0972	149.0966	−4.0	131, 105	WS, LG
146	Rosmanol	C_20_H_26_O_5_	66.950	[M−H]−	345.1707	345.1716	2.6	301	LG, WS, OSB
**Tyrosols**									
147	Hydroxytyrosol	C_8_H_10_O_3_	17.703	** [M−H]−	153.0557	153.0557	0.0	123, 109	LG, WS
148	Oleoside dimethylester	C_18_H_26_O_11_	24.097	[M−H]−	417.1402	417.1407	1.2	255, 223	LG
149	Oleoside 11-methylester	C_17_H_24_O_11_	25.331	[M−H]−	403.1246	403.1229	−4.2	223, 165	LG, OSB
150	Hydroxytyrosol 4-*O*-glucoside	C_14_H_20_O_8_	65.148	[M+H]+	317.1231	317.1240	2.8	153, 123	OSB, WS
**Other polyphenols**									
151	* Pyrogallol	C_6_H_6_O_3_	6.821	[M−H]−	125.0244	125.0242	−1.6	107, 97, 79	LG, WS, OSB
152	Catechol	C_6_H_6_O_2_	6.927	[M−H]−	109.0295	109.0295	0.0	65	LG, SWN
153	Salvianolic acid B	C_36_H_30_O_16_	32.818	[M−H]−	717.1461	717.1497	5.0	520, 357, 179, 161	SWN
154	Phlorin	C_12_H_16_O_8_	63.601	[M+H]+	289.0918	289.0907	−3.8	125	LG
155	Salvianolic acid G	C_20_H_18_O_10_	69.870	[M+H]+	419.0973	419.0993	4.8	399, 237, 219, 179	LG

* = compounds were identified through pure standards; ** = compounds were identified in both modes (positive and negative); lemongrass (LG), sandalwood nuts (SWN), wattle seeds (WS) and old man saltbush (OSB).

## Data Availability

Not applicable.

## Supplementary Materials

The following supporting information can be downloaded at: https://www.mdpi.com/article/10.3390/metabo12111016/s1, Figure S1: Base Peak Chromatograms (BPC) of Australian native lemongrass, old man saltbush, wattle seeds, and sandalwood nuts in positive (black) and negative (blue) modes; Figure S2: Chromatograms and mass spectra of some selected compounds; Table S1: Antioxidant activities of Australian native plants; Table S2: Semi-quantification of phytochemical metabolites in Australian native plants (μg/g).
