# Supplementary material for: LC-MS/MS Characterization of Phenolic Metabolites and Their Antioxidant Activities from Australian Native Plants
Source: Metabolites. 2022 Oct 24;12(11):1016. doi: 10.3390/metabo12111016 (PMC9698446; doi:10.3390/metabo12111016)
Supplement: Supplementary file 1 [file metabolites-12-01016-s001.zip › metabolites-1967583-supplementary.pdf]

Supplementary Material

LC-MS/MS Characterization of Phenolic Metabolites and Their Antioxidant Activities from Australian Native Plants

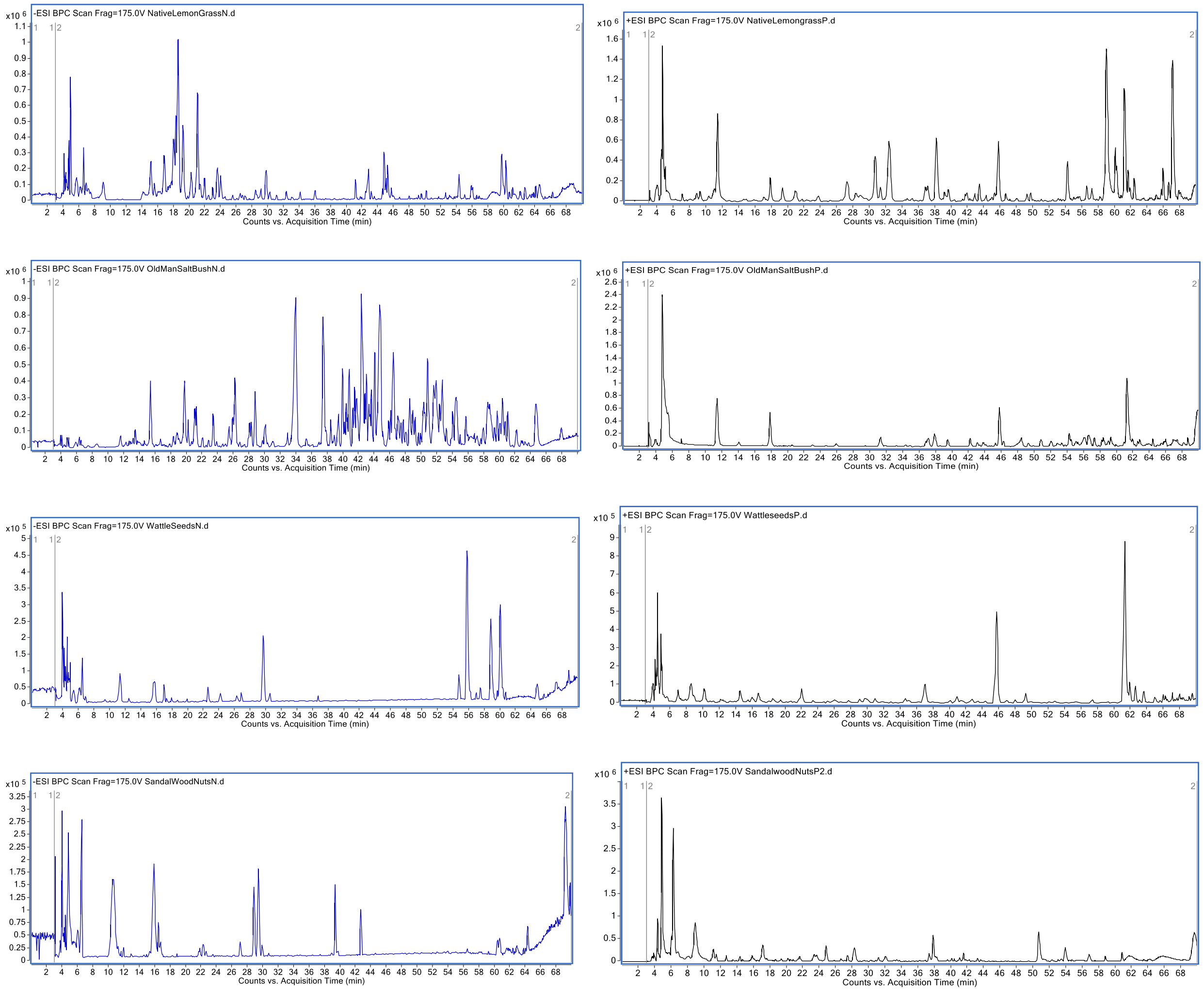

Figure S1. Base Peak Chromatograms (BPC) of Australian native lemon grass, Oldman saltbush, wattle seeds, and sandalwood nuts in positive (black) and negative (blue) modes.

Compound 1

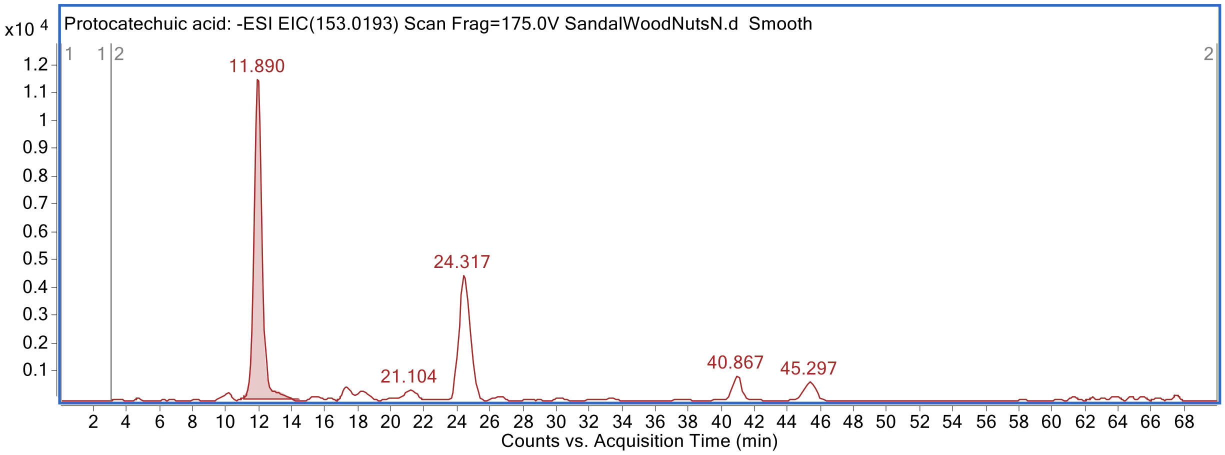
x10 4
Protocatechuic acid: -ESI EIC(153.0193) Scan Frag=175.0V SandalWoodNutsN.d Smooth
11.890
24.317
21.104
40.867
45.297
Counts vs. Acquisition Time (min)

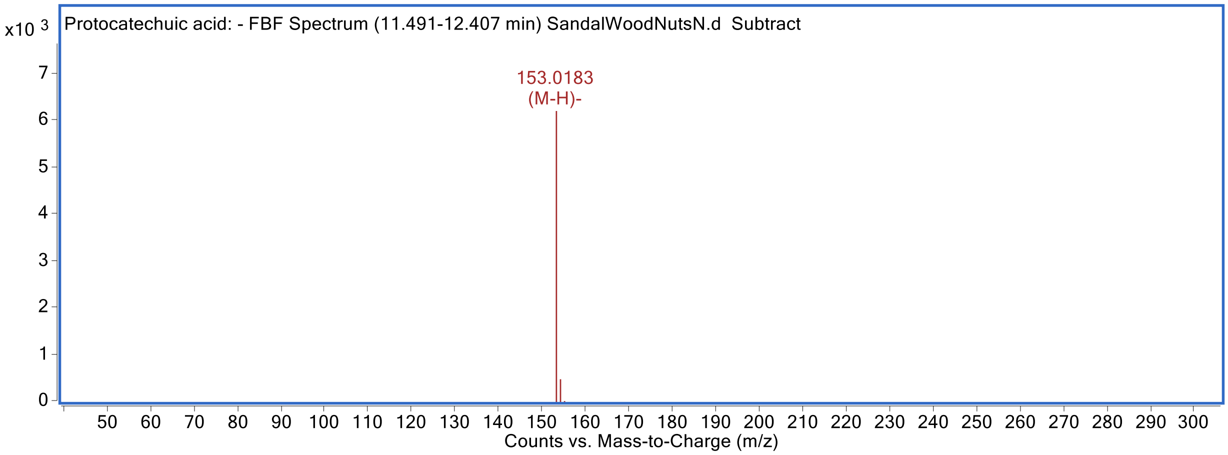
x10 3
Protocatechuic acid: - FBF Spectrum (11.491-12.407 min) SandalWoodNutsN.d Subtract
153.0183
(M-H)-
Counts vs. Mass-to-Charge (m/z)

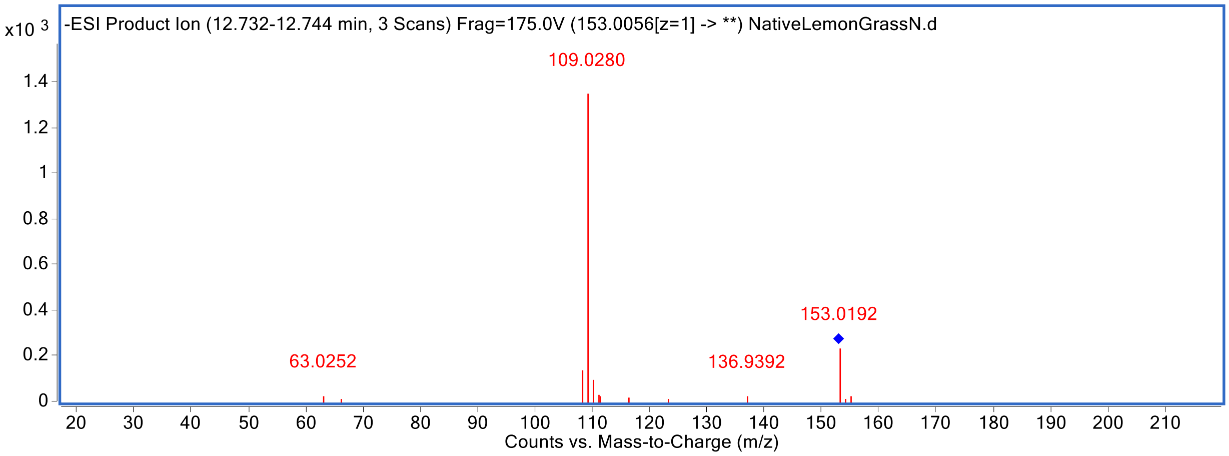
x10 3
-ESI Product Ion (12.732-12.744 min, 3 Scans) Frag=175.0V (153.0056[z=1] -> **) NativeLemonGrassN.d
109.0280
153.0192
63.0252
136.9392
Counts vs. Mass-to-Charge (m/z)

Compound 4

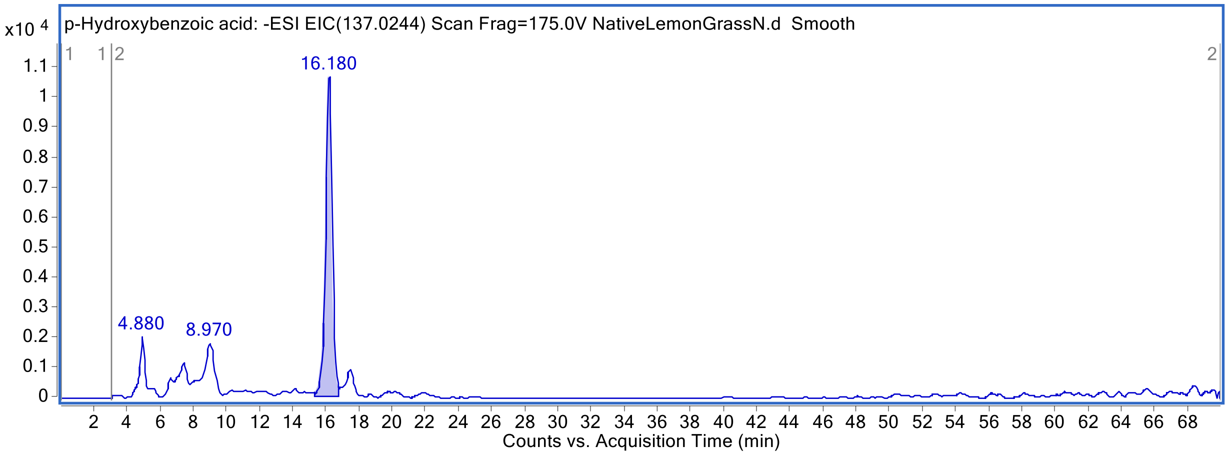

x10 4
p-Hydroxybenzoic acid: -ESI EIC(137.0244) Scan Frag=175.0V NativeLemonGrassN.d Smooth
16.180
4.880
8.970
Counts vs. Acquisition Time (min)

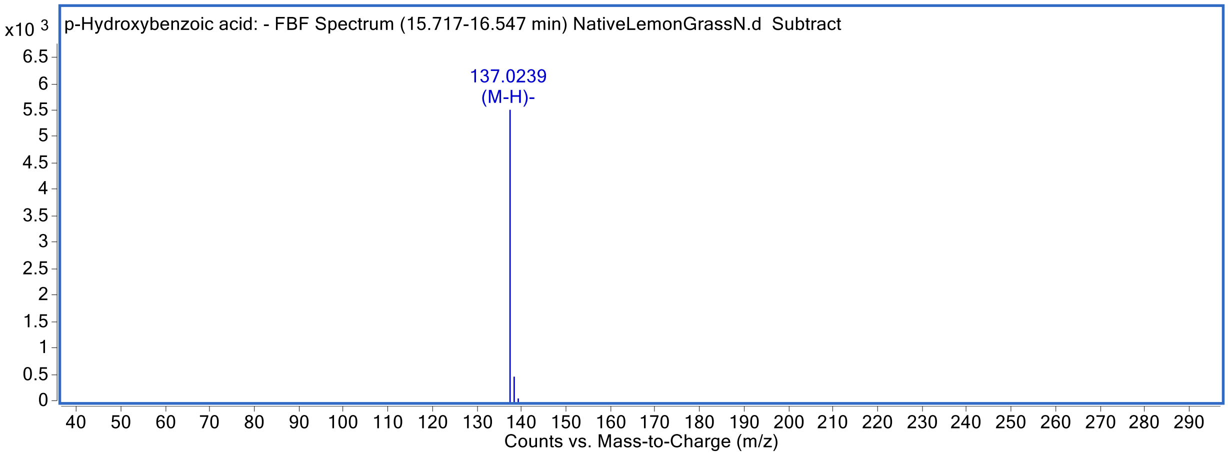

x10 3
p-Hydroxybenzoic acid: - FBF Spectrum (15.717-16.547 min) NativeLemonGrassN.d Subtract
137.0239
(M-H)-
Counts vs. Mass-to-Charge (m/z)

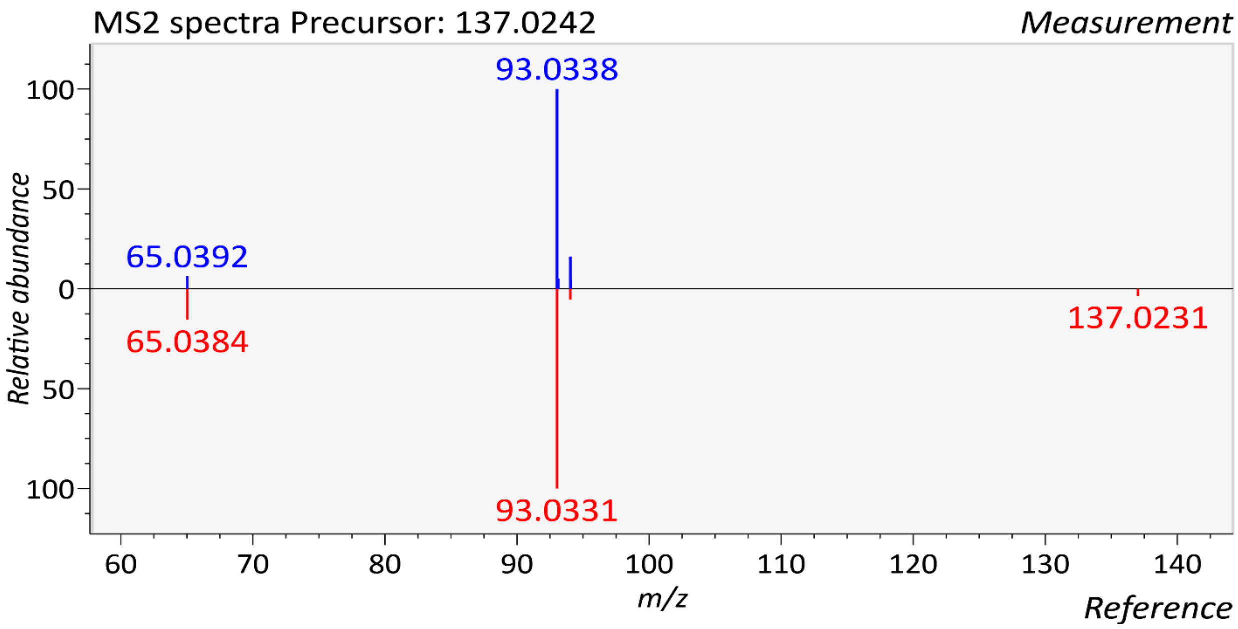

MS2 spectra Precursor: 137.0242
Measurement
93.0338
65.0392
65.0384
137.0231
93.0331
Relative abundance
m/z
Reference

Compound 16

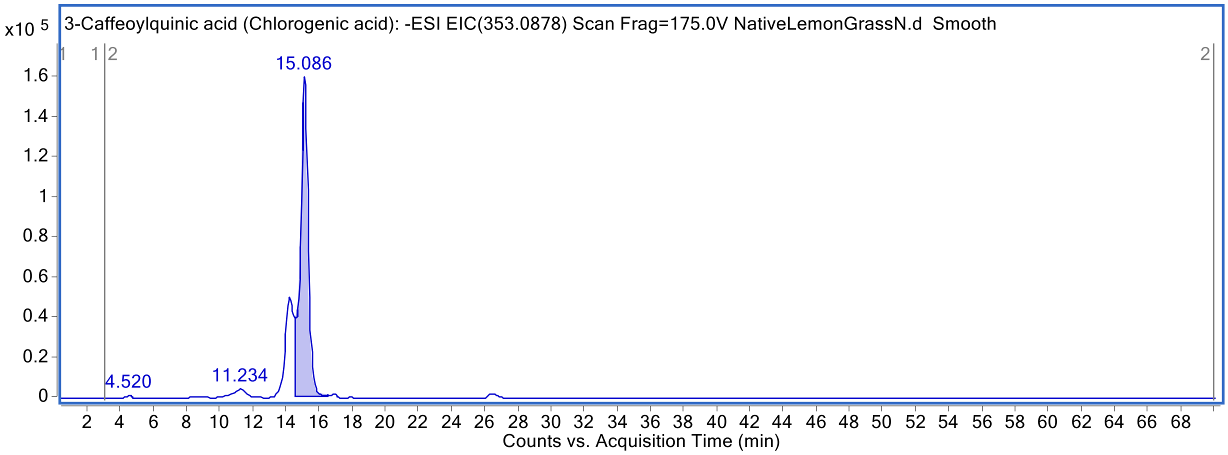

3-Caffeoylquinic acid (Chlorogenic acid): -ESI EIC(353.0878) Scan Frag=175.0V NativeLemonGrassN.d Smooth
x10 5
15.086
11.234
4.520
Counts vs. Acquisition Time (min)

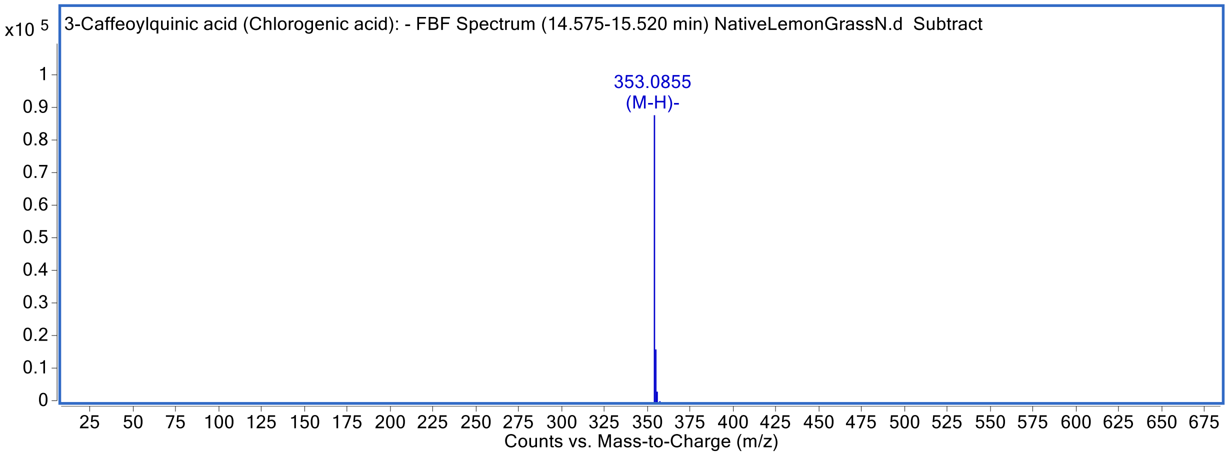

3-Caffeoylquinic acid (Chlorogenic acid): - FBF Spectrum (14.575-15.520 min) NativeLemonGrassN.d Subtract
x10 5
353.0855
(M-H)-
Counts vs. Mass-to-Charge (m/z)

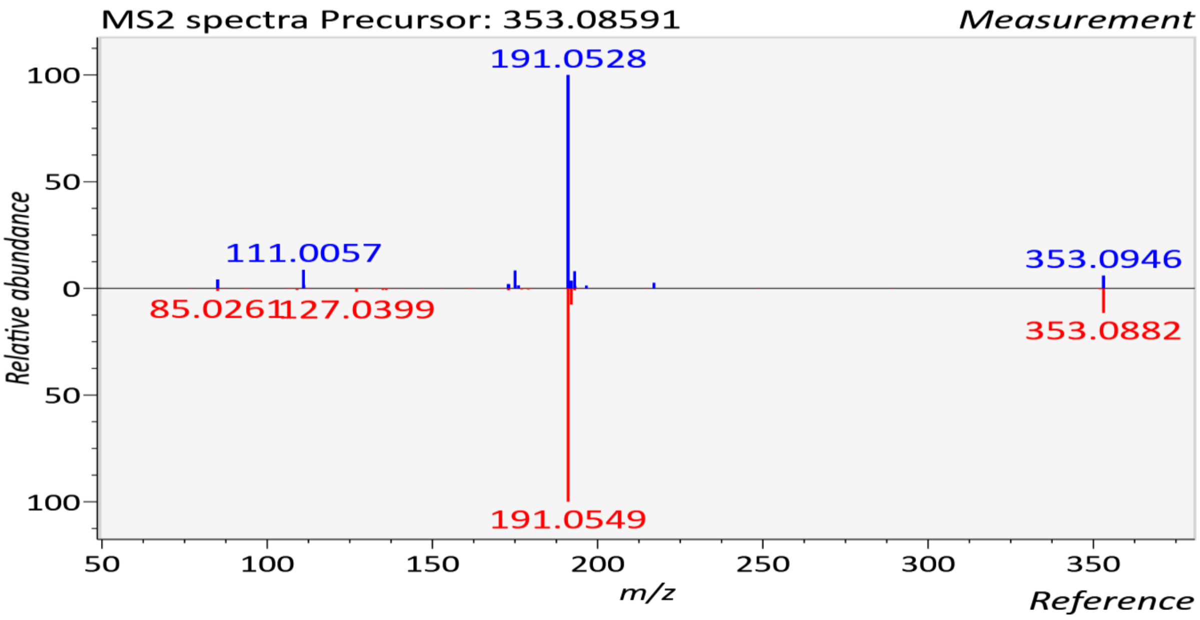

MS2 spectra Precursor: 353.08591
Measurement
191.0528
111.0057
353.0946
85.0261
127.0399
353.0882
191.0549
Relative abundance
m/z
Reference

Compound 18

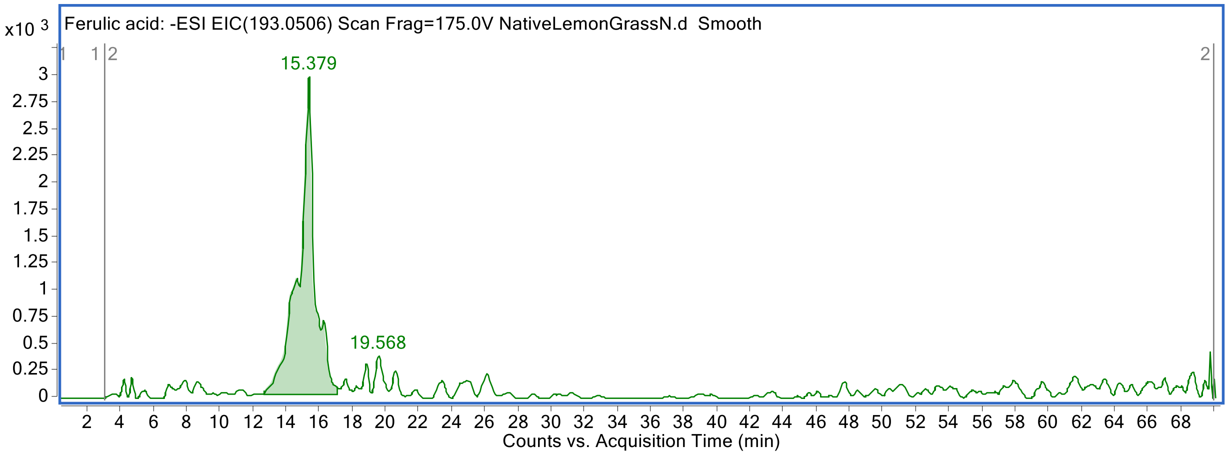
x10 3
Ferulic acid: -ESI EIC(193.0506) Scan Frag=175.0V NativeLemonGrassN.d Smooth
15.379
19.568
Counts vs. Acquisition Time (min)

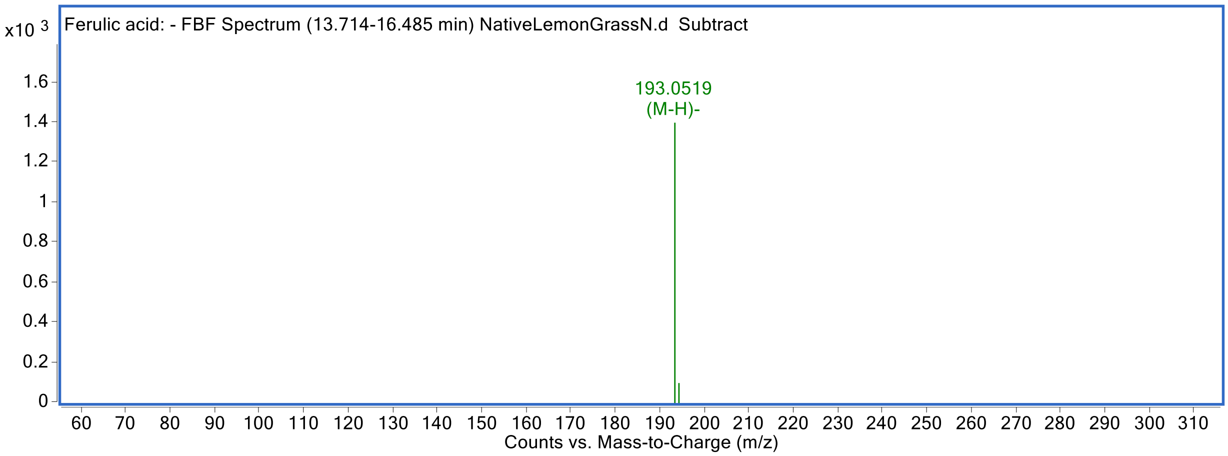
x10 3
Ferulic acid: - FBF Spectrum (13.714-16.485 min) NativeLemonGrassN.d Subtract
193.0519
(M-H)-
Counts vs. Mass-to-Charge (m/z)

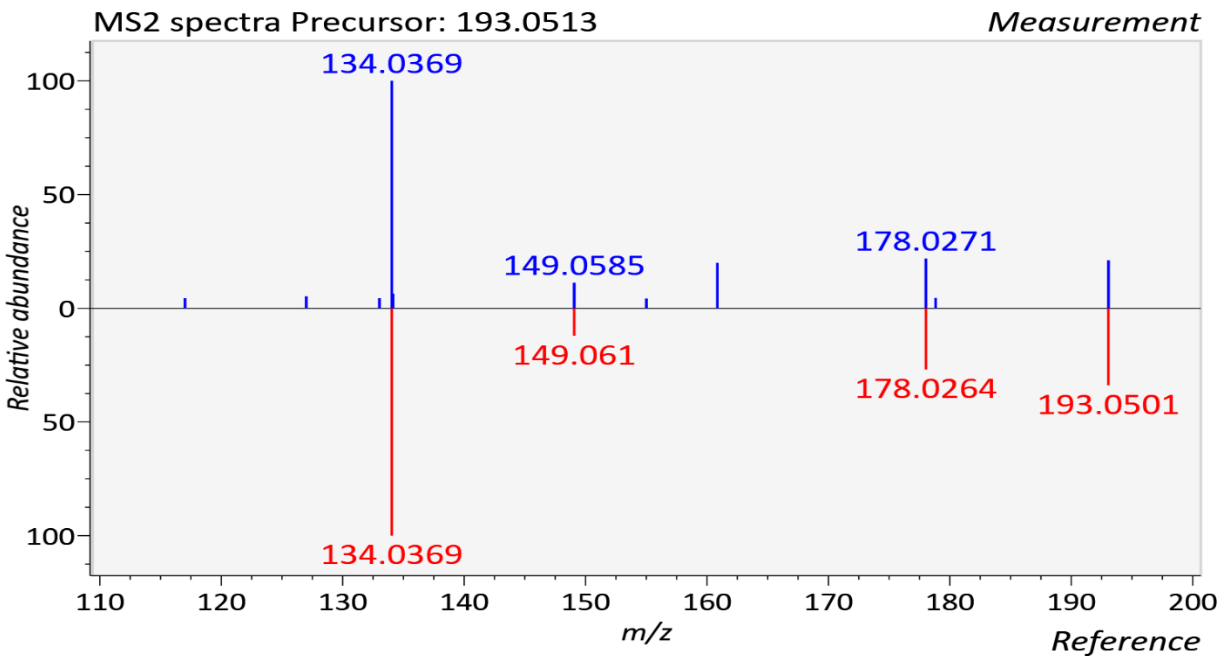
MS2 spectra Precursor: 193.0513
Measurement
134.0369
149.0585
178.0271
149.061
178.0264
193.0501
134.0369
Relative abundance
m/z
Reference

Compound 21

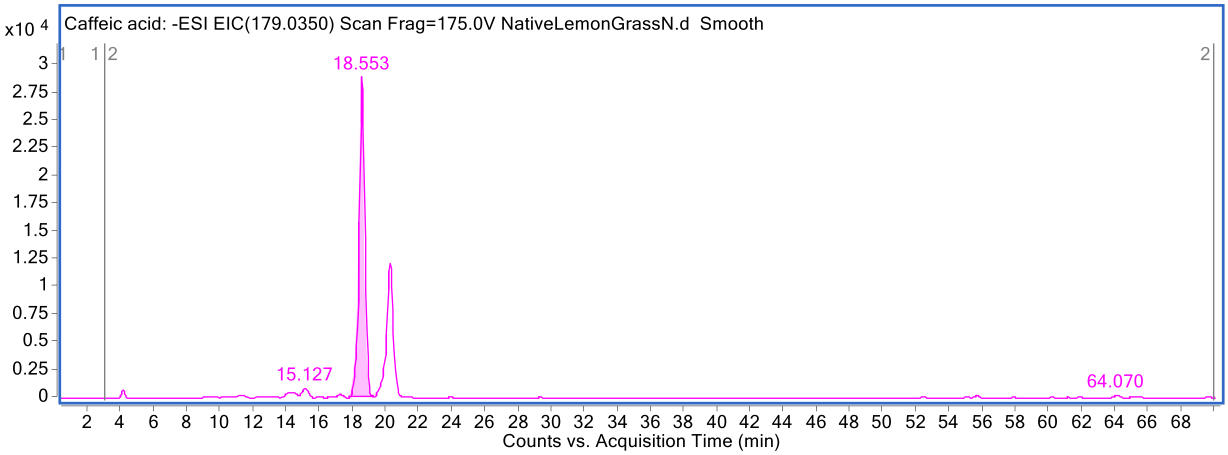
Caffeic acid: -ESI EIC(179.0350) Scan Frag=175.0V NativeLemonGrassN.d Smooth
x10 4
18.553
15.127
64.070
Counts vs. Acquisition Time (min)

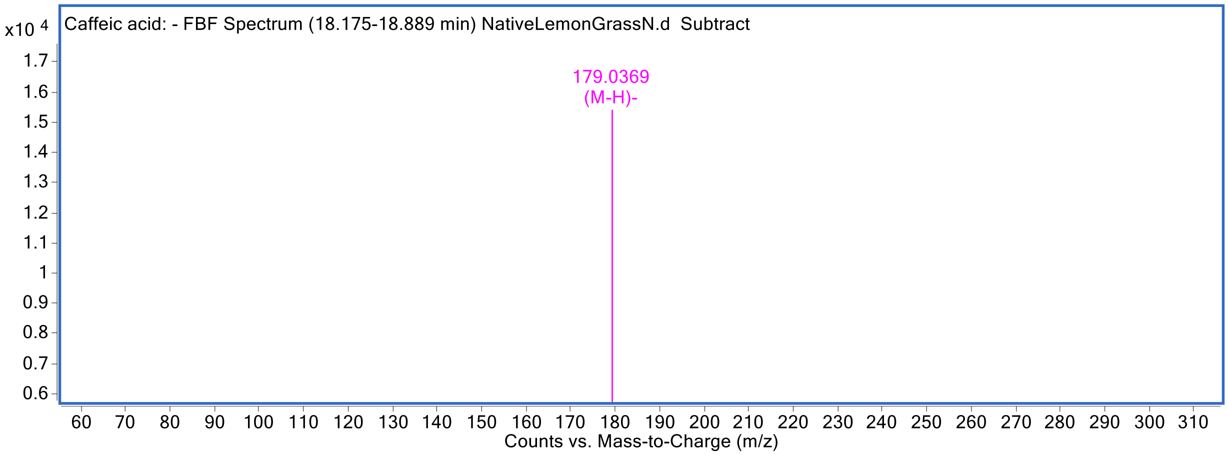
Caffeic acid: - FBF Spectrum (18.175-18.889 min) NativeLemonGrassN.d Subtract
x10 4
179.0369
(M-H)-
Counts vs. Mass-to-Charge (m/z)

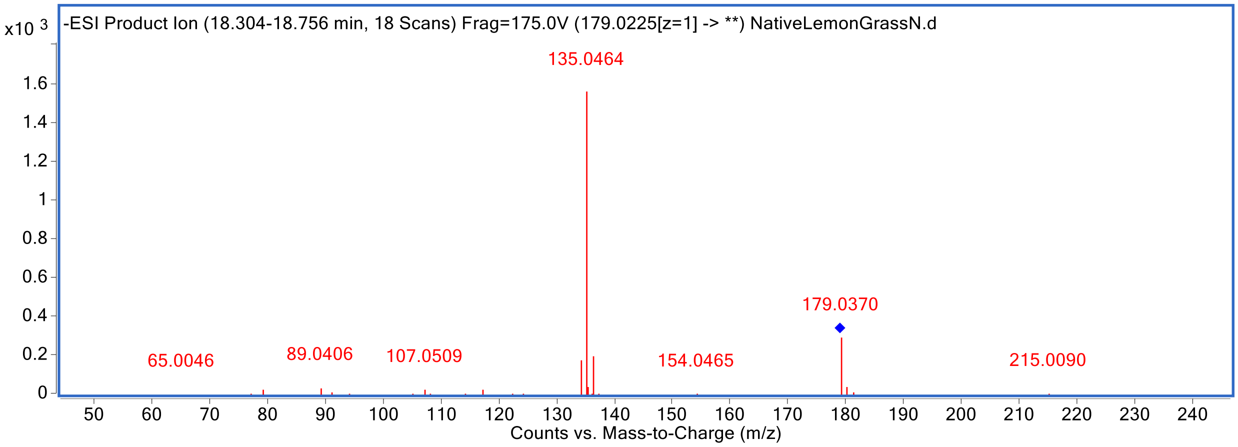
-ESI Product Ion (18.304-18.756 min, 18 Scans) Frag=175.0V (179.0225[z=1] -> **) NativeLemonGrassN.d
x10 3
135.0464
179.0370
65.0046
89.0406
107.0509
154.0465
215.0090
Counts vs. Mass-to-Charge (m/z)

Compound 22

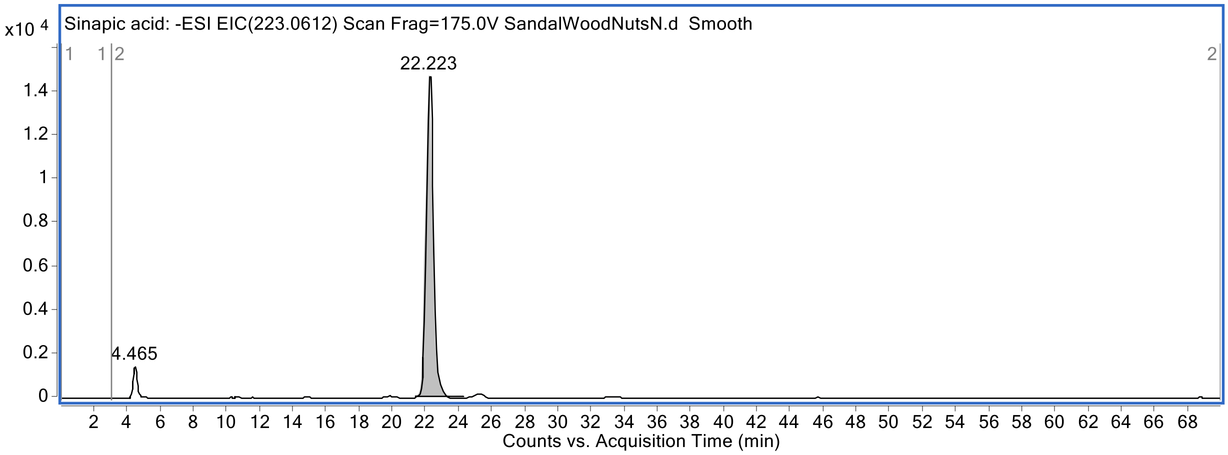
Sinapic acid: -ESI EIC(223.0612) Scan Frag=175.0V SandalWoodNutsN.d Smooth
x10 4
22.223
4.465
Counts vs. Acquisition Time (min)

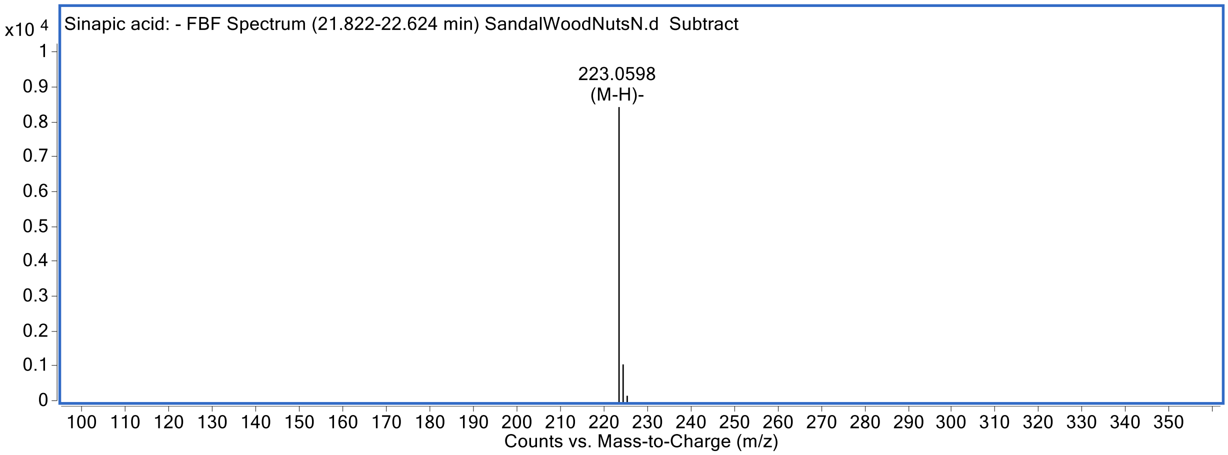
Sinapic acid: - FBF Spectrum (21.822-22.624 min) SandalWoodNutsN.d Subtract
x10 4
223.0598
(M-H)-
Counts vs. Mass-to-Charge (m/z)

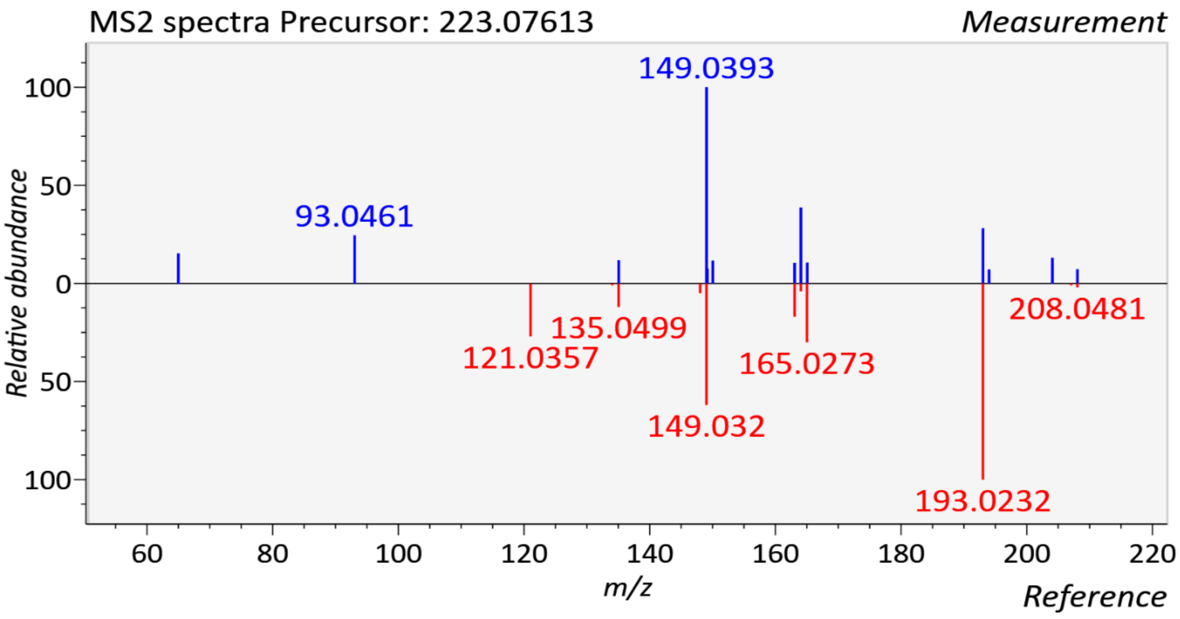
MS2 spectra Precursor: 223.07613
Measurement
149.0393
93.0461
135.0499
121.0357
165.0273
149.032
208.0481
193.0232
Relative abundance
m/z
Reference

Compound 23

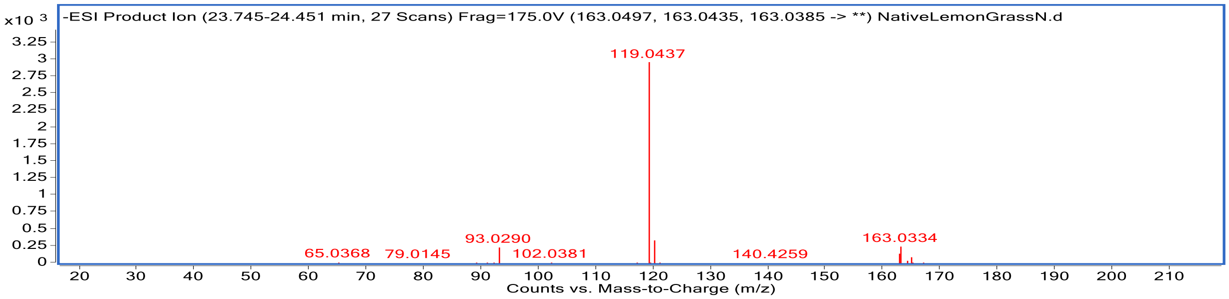

Compound 28

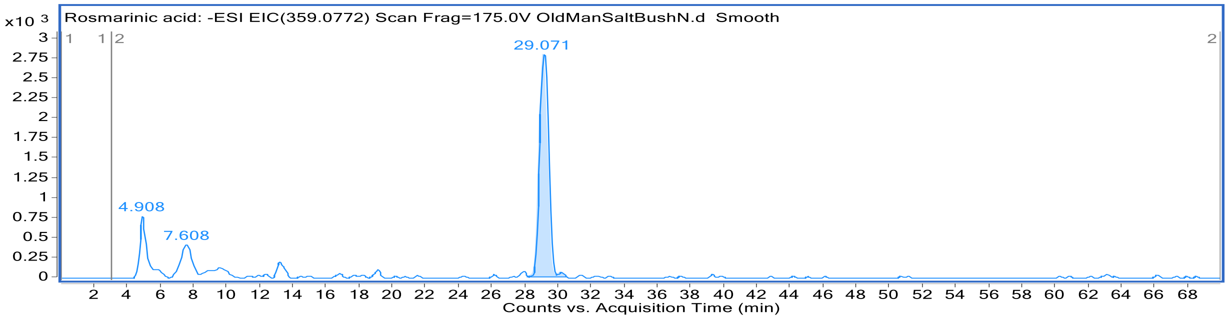

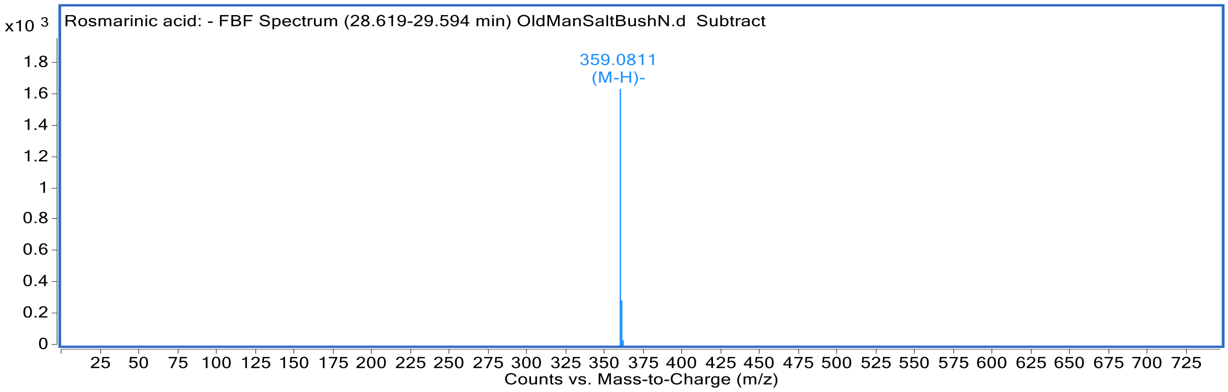

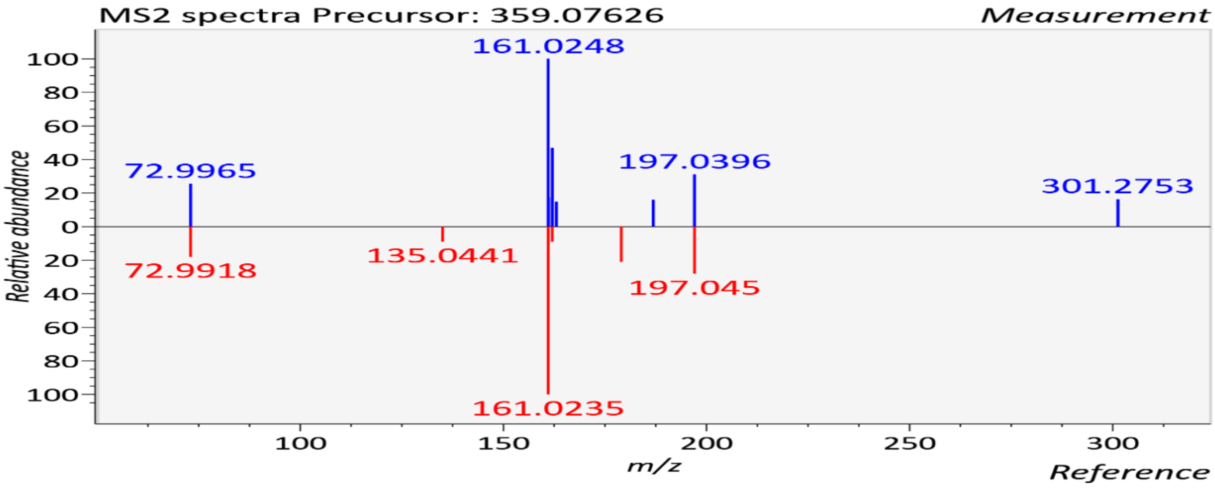

Compound 42

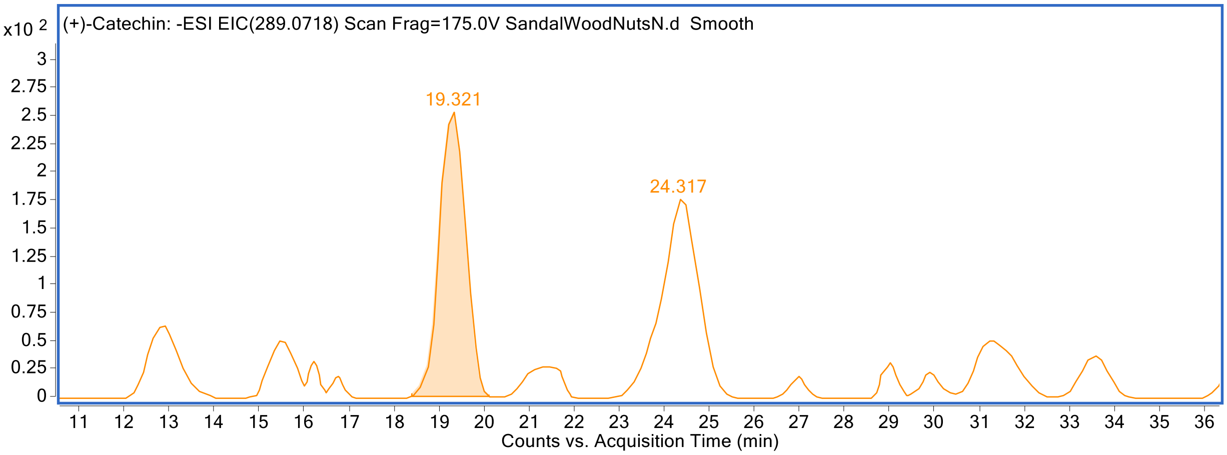
x10 2
(+)-Catechin: -ESI EIC(289.0718) Scan Frag=175.0V SandalWoodNutsN.d Smooth
19.321
24.317
Counts vs. Acquisition Time (min)

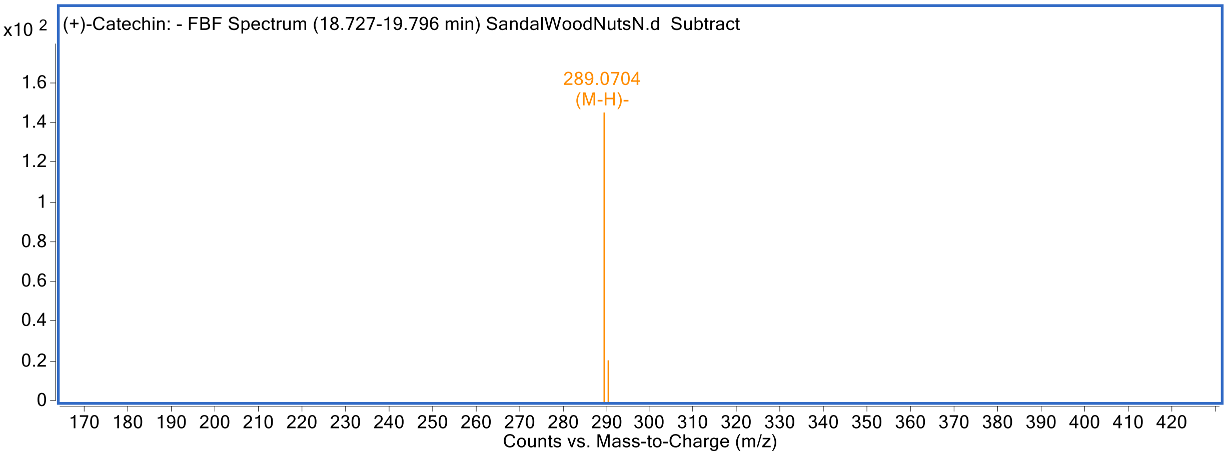
x10 2
(+)-Catechin: - FBF Spectrum (18.727-19.796 min) SandalWoodNutsN.d Subtract
289.0704
(M-H)-
Counts vs. Mass-to-Charge (m/z)

Compound 46

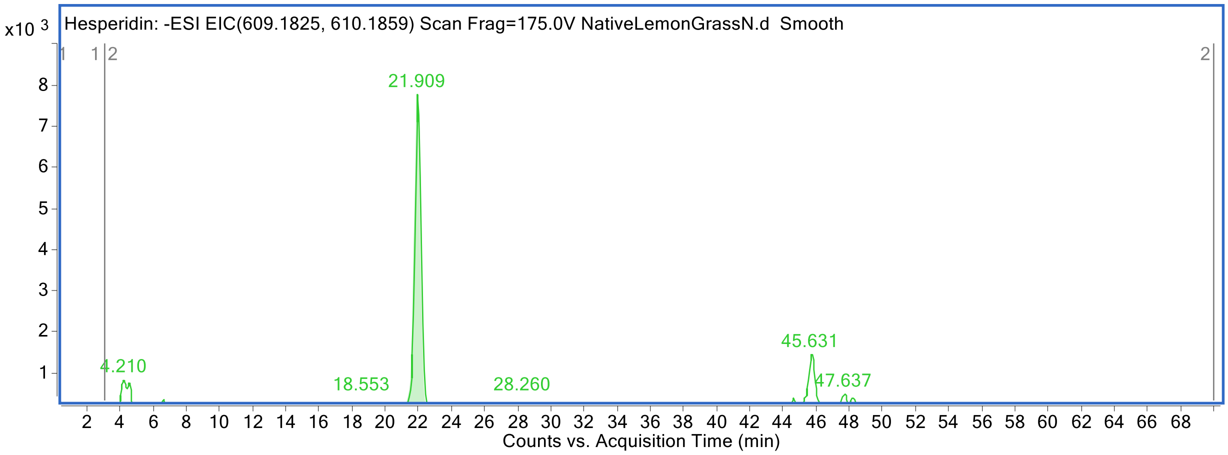

x10 3
Hesperidin: -ESI EIC(609.1825, 610.1859) Scan Frag=175.0V NativeLemonGrassN.d Smooth
21.909
4.210
18.553
28.260
45.631
47.637
Counts vs. Acquisition Time (min)

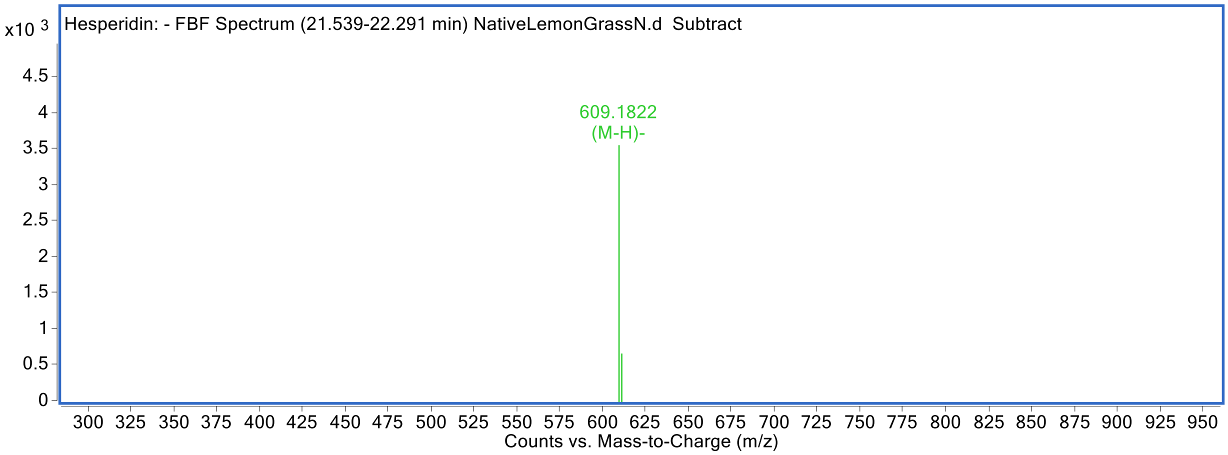

x10 3
Hesperidin: - FBF Spectrum (21.539-22.291 min) NativeLemonGrassN.d Subtract
609.1822
(M-H)-
Counts vs. Mass-to-Charge (m/z)

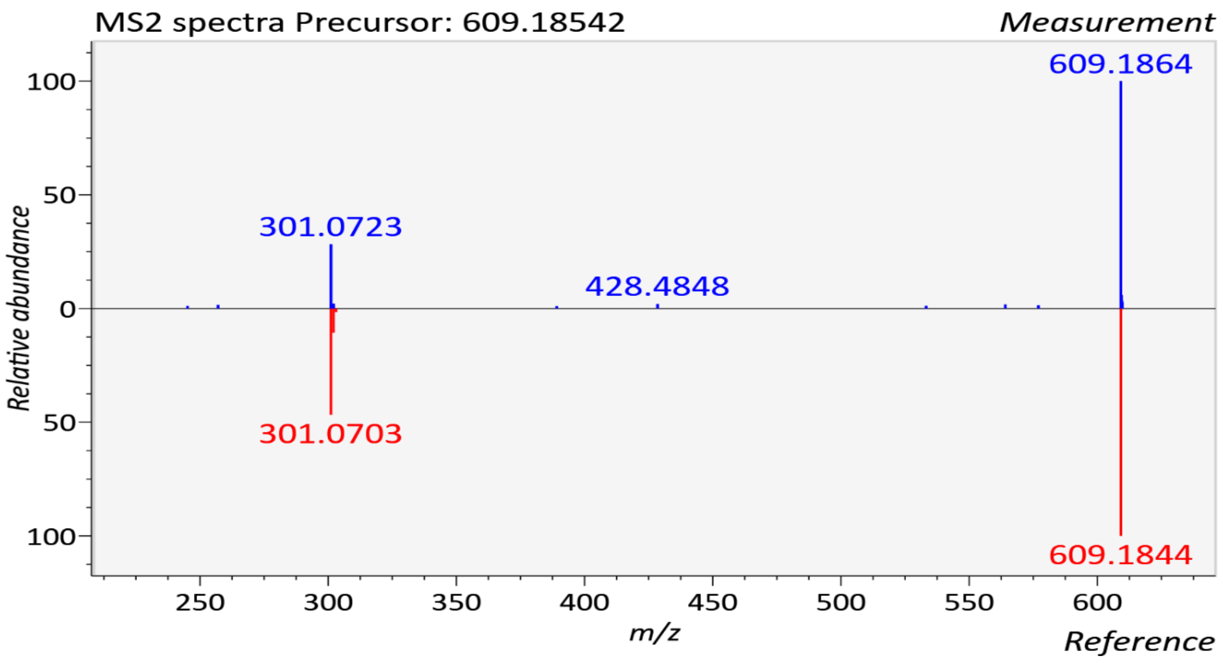

MS2 spectra Precursor: 609.18542
Measurement
609.1864
301.0723
428.4848
301.0703
609.1844
Relative abundance
m/z
Reference

Compound 57

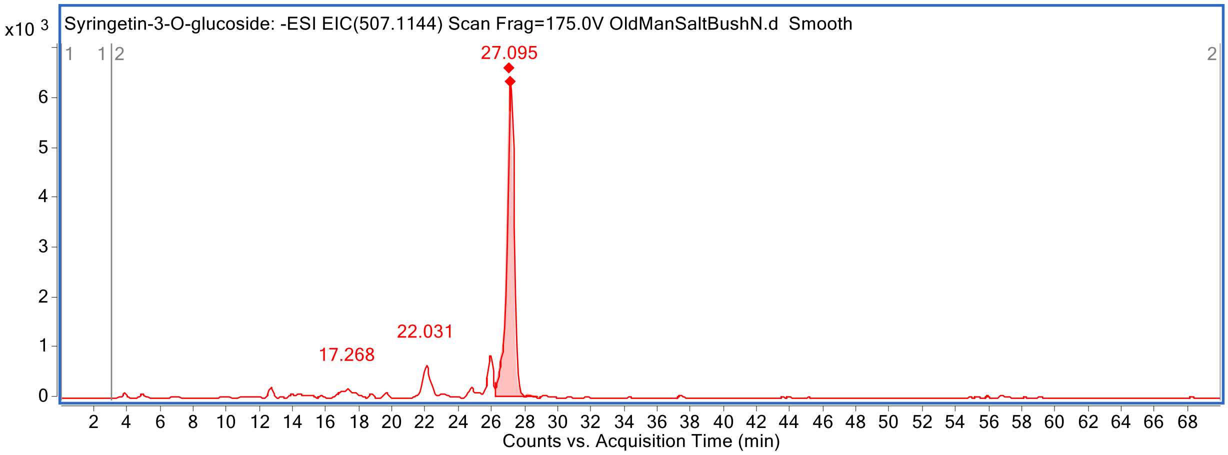

x10 3
Syringetin-3-O-glucoside: -ESI EIC(507.1144) Scan Frag=175.0V OldManSaltBushN.d Smooth
27.095
22.031
17.268
Counts vs. Acquisition Time (min)

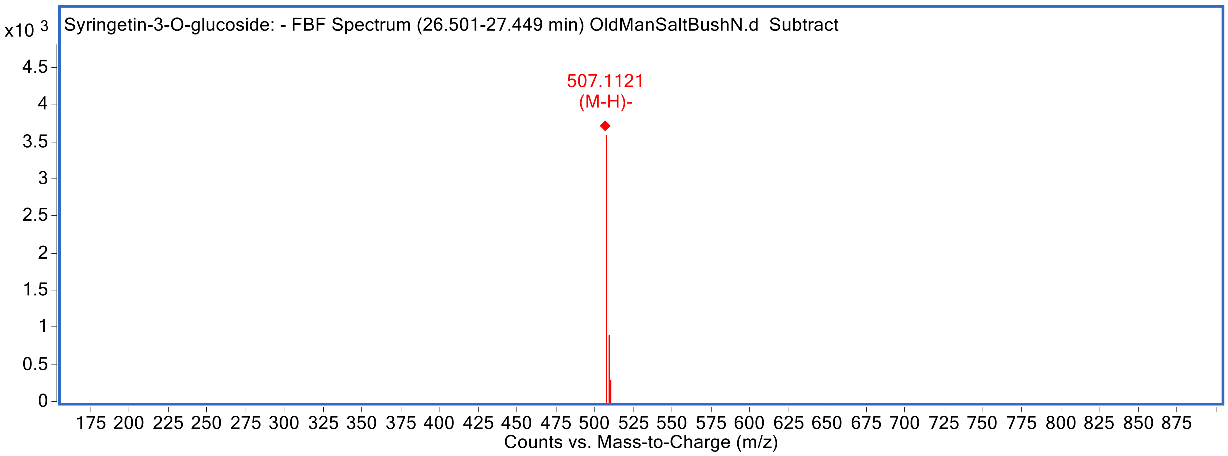

x10 3
Syringetin-3-O-glucoside: - FBF Spectrum (26.501-27.449 min) OldManSaltBushN.d Subtract
507.1121
(M-H)-
Counts vs. Mass-to-Charge (m/z)

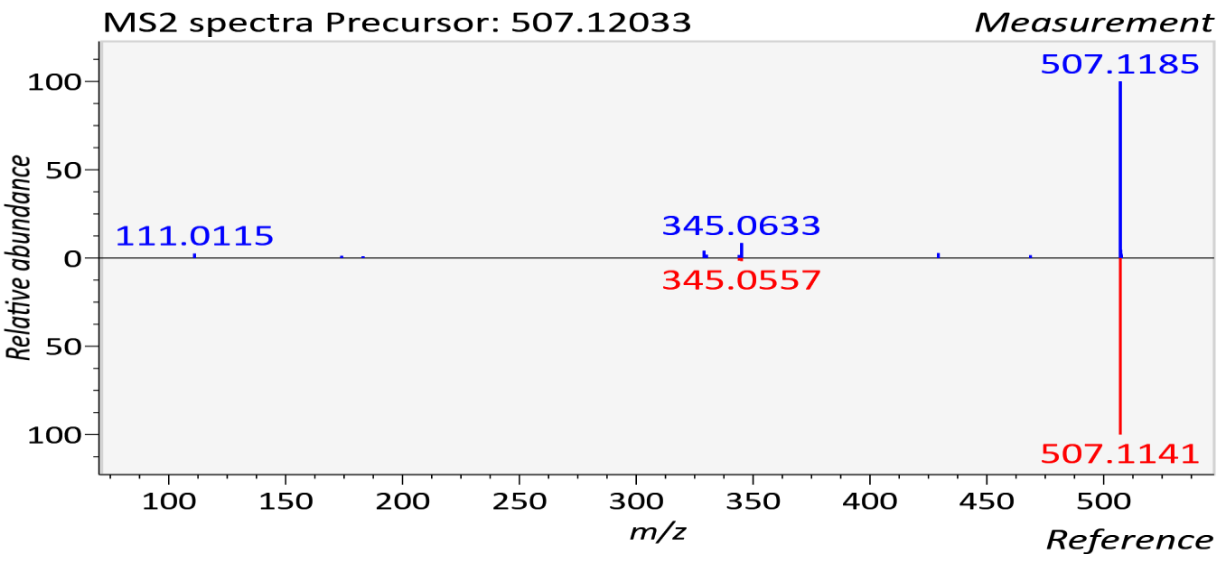

MS2 spectra Precursor: 507.12033
Measurement
507.1185
111.0115
345.0633
345.0557
507.1141
Relative abundance
m/z
Reference

Compound 65

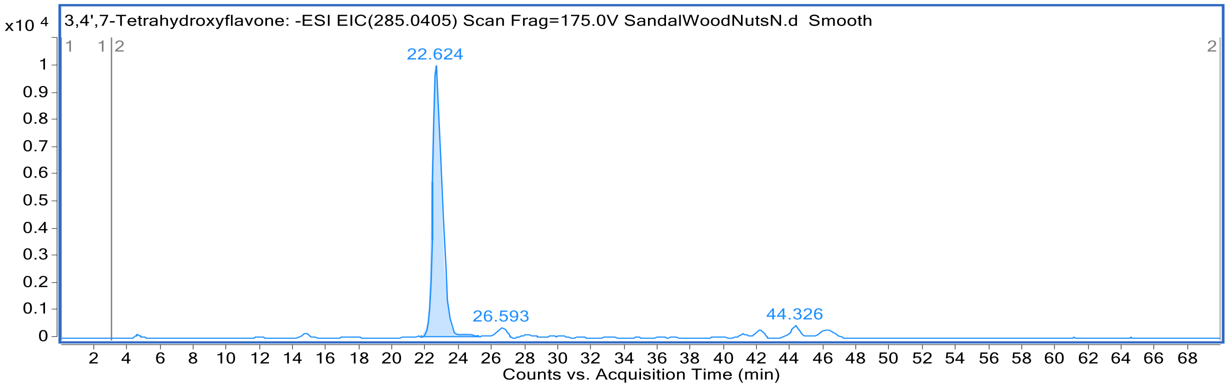

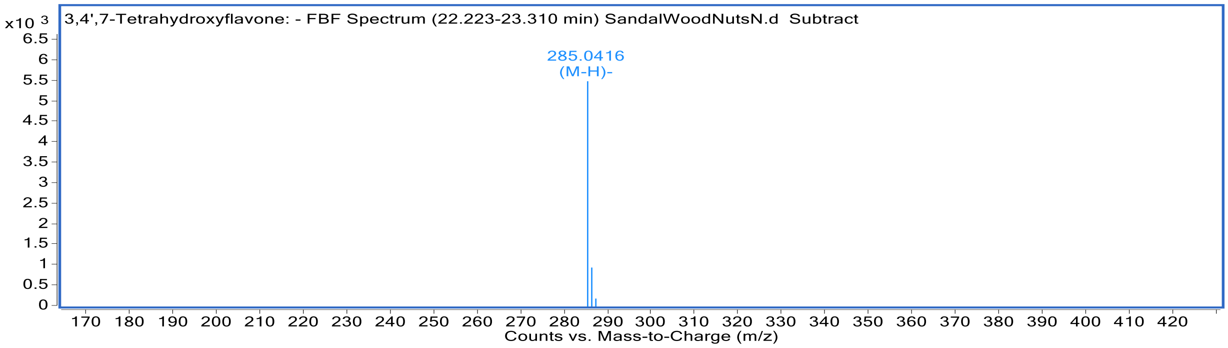

Compound 125

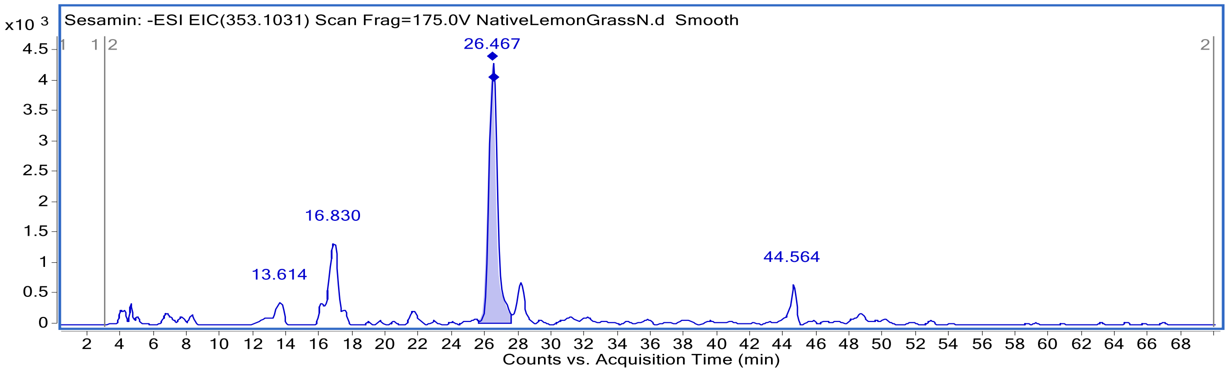

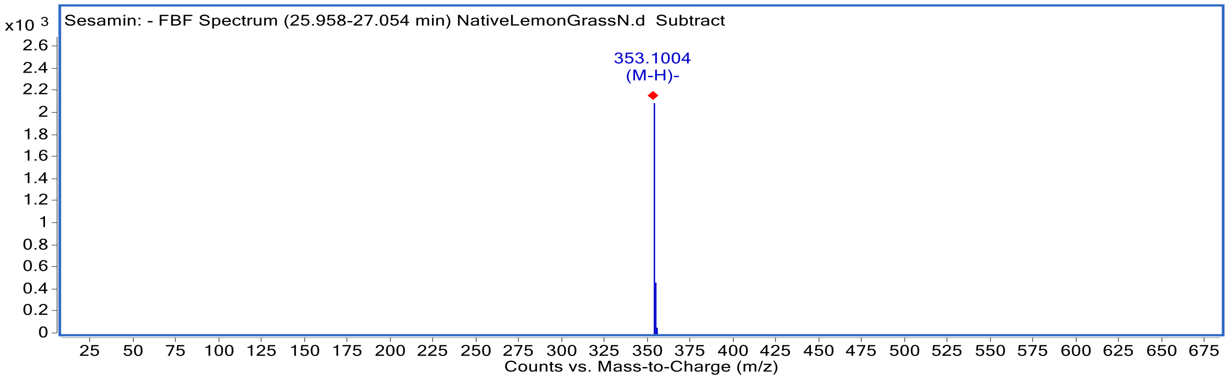

Compound 138

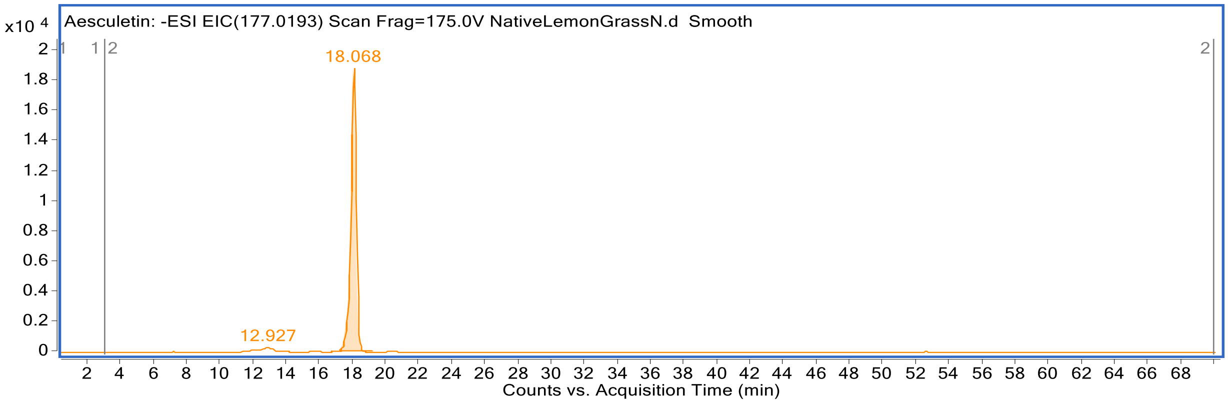

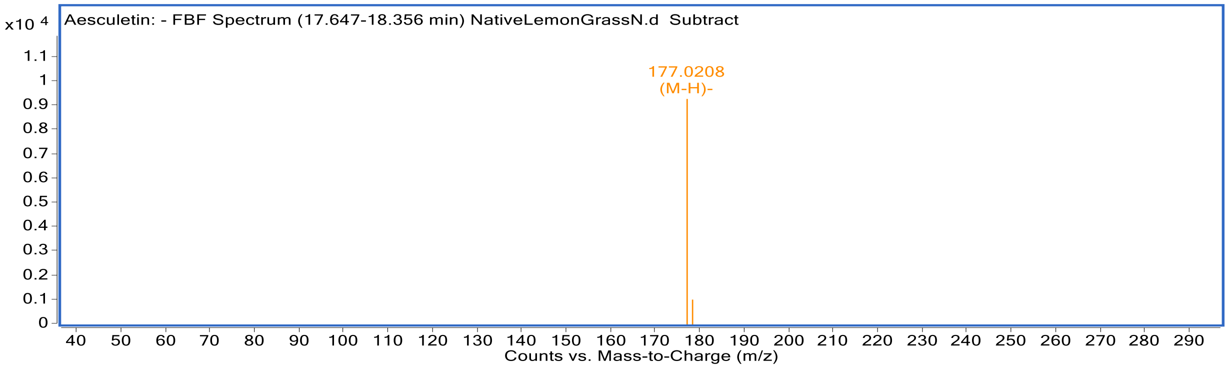

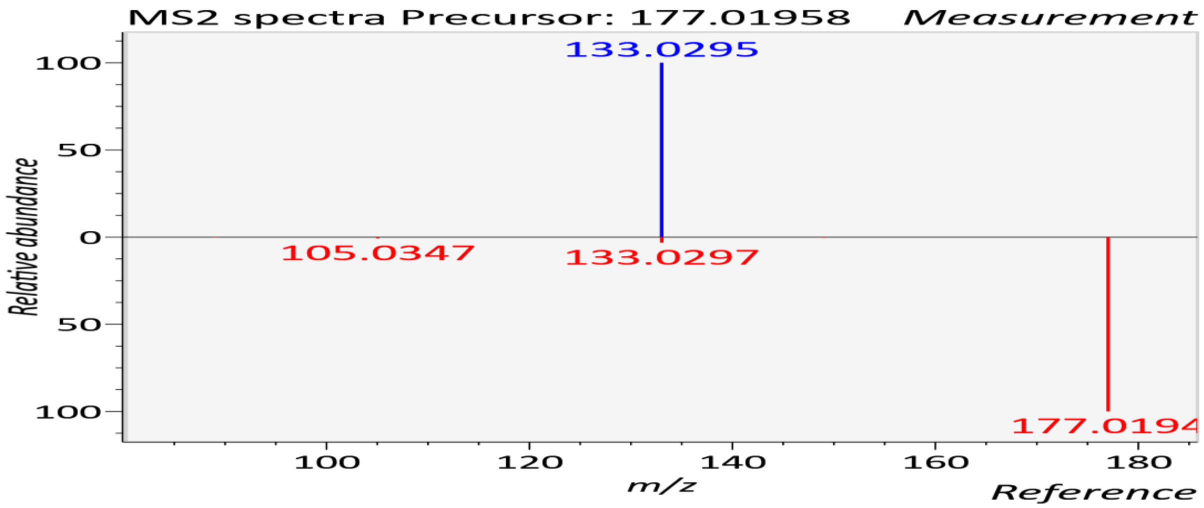

Compound 145

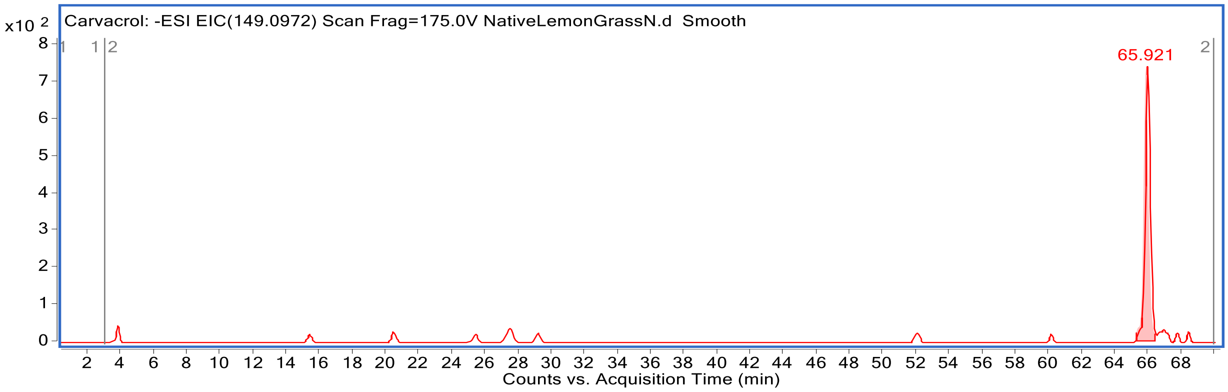

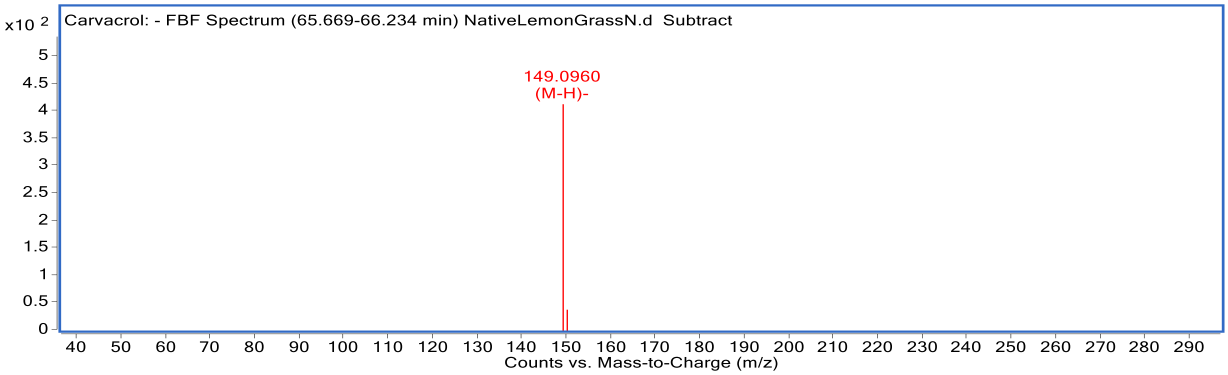

Compound 151

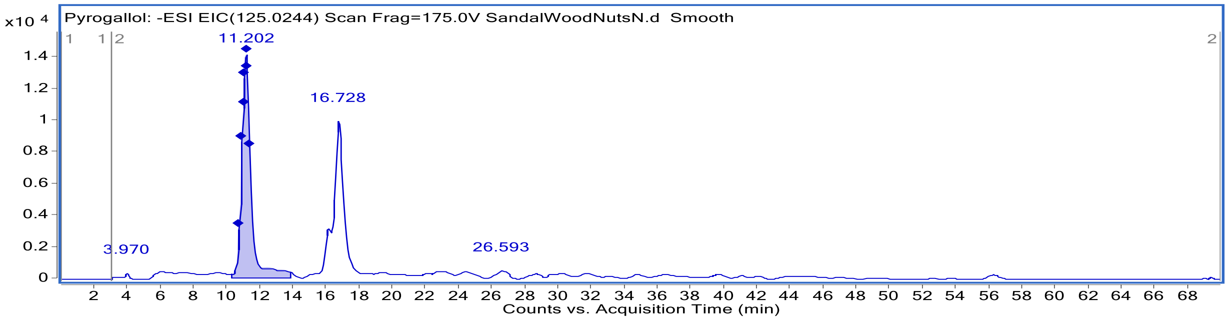

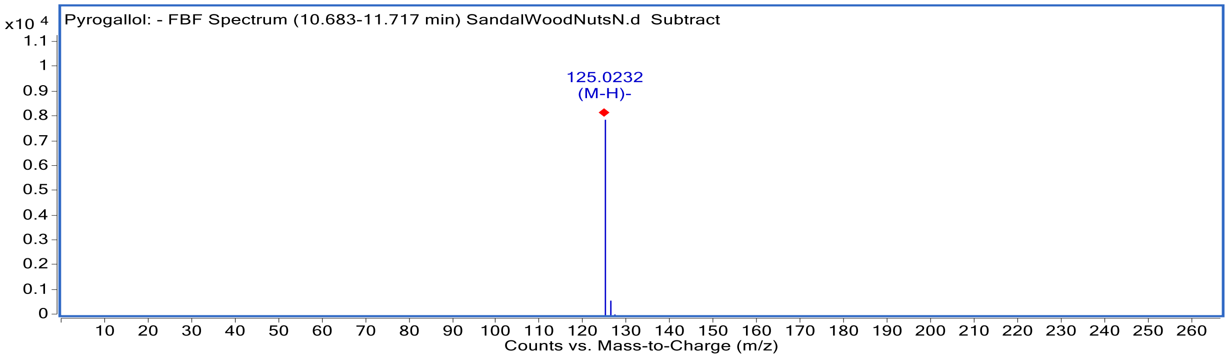

Figure S2. Chromatograms and mass spectra of some selected compounds

Table S1. Antioxidant activities of Australian native plants

Variables	DPPH (mg AAE/g)	ABTS (mg AAE/g)	FRAP (mg AAE/g)	•OH-RSA (mg AAE/g)	FICA (mg EDTA/g)	PMA (mg AAE/g)
Sandalwood Nuts	10.30 ± 0.9 [b]	37.72 ± 1.40 [d]	19.48 ± 3.04 [a]	47.25 ± 0.95 [b]	1.56 ± 0.24 [b]	14.43 ± 1.86 [a]
Lemongrass	18.73 ± 2.8 [a]	98.81 ± 6.19 [a]	14.55 ± 1.32 [b]	104.34 ± 6.92 [a]	2.48 ± 0.16 [a]	12.88 ± 0.64 [a]
Oldman Salt Bush	9.57 ± 0.1 [b]	74.76 ± 1.61 [b]	5.62 ± 1.19 [c]	47.78 ± 0.35 [b]	1.50 ± 0.14 [b]	10.88 ± 0.53 [c]
Wattle Seeds	8.03 ± 0.3 [bc]	56.90 ± 2.90 [c]	2.52 ± 1.97 [d]	19.25 ± 0.92 [c]	1.39 ± 0.03 [b]	10.78 ± 0.69 [c]

2,2'-azinobis-(3-ethylbenzothiazoline-6-sulfonic acid (ABTS), ferric reducing antioxidant power (FRAP), hydroxyl-radical scavenging activity (•OH-RSA), ferrous ion chelating assay (FICA), 2,2'-diphenyl-1-picrylhydrazyl (DPPH) and phosphomolybdate assay (PMA). Values (mean ± standard deviation; n=3) within the column are significantly different ($p < 0.05$) from each other represented by the superscript letters (a-d).

Table S2. Semi-quantification of phytochemical metabolites in Australian native plants (μg/g).

No.	Compounds	LG	WS	OSB	SWN
1	Diosmin	19.32 ± 5.47	11.04 ± 2.14		
2	Catechin	19.23 ± 2.37	11.54 ± 3.07		
3	Procyanidin B2	46.75 ± 6.56			
4	Quercetin-3-glucoside	151.35 ± 11.34	26.43 ± 4.23		
5	Tricin	12.34 ± 2.31			
6	Kaempferol-3-glucoside	21.45 ± 4.12			
7	Caffeic acid	445.21 ± 32.77			78.95 ± 1.98
8	Chlorogenic acid	377.65 ± 4.26	36.98 ± 6.91	18.76 ± 6.34	29.32 ± 5.86
9	Cinnamic acid	61.30 ± 17.31			
10	Quinic acid	161.52 ± 17.62	39.64 ± 9.92	19.64 ± 3.92	
11	Sinapic acid				77.17 ± 6.85
12	Syringic acid				17.04 ± 3.45
13	Ferulic acid	12.17 ± 3.11			
14	Gallic Acid		18.53 ± 6.15		93.32 ± 18.44
15	*p*-Coumaric acid	393.32 ± 39.56			58.63 ± 4.12
16	*p*-Hydroxybenzoic acid	94.01 ± 2.24			30.11 ± 3.43
17	Protocatechuic acid	54.16 ± 3.65			91.61 ± 3.72
18	Pyrogallol	27.54 ± 5.76	18.59 ± 4.17	11.02 ± 1.63	

Lemon grass (LG), Wattle Seeds (WS), Oldman saltbush (OSB), Sandalwood Nuts (SWN)
